# Gene Expression Profiling of Embryonic Human Neural Stem Cells and Dopaminergic Neurons from Adult Human Substantia Nigra

**DOI:** 10.1371/journal.pone.0028420

**Published:** 2011-12-07

**Authors:** Hany E. S. Marei, Asma Althani, Nahla Afifi, Fabrizio Michetti, Mario Pescatori, Roberto Pallini, Patricia Casalbore, Carlo Cenciarelli, Philip Schwartz, Abd-Elmaksoud Ahmed

**Affiliations:** 1 Department of Cytology and Histology, Faculty of Veterinary Medicine, Mansoura University, Mansoura, Egypt; 2 Health Sciences Department, College of Arts and Sciences, Qatar University, Doha, Qatar; 3 Institute of Anatomy and Cell Biology, Università Cattolica del S. Cuore, Roma, Italy; 4 Institute of Neurosurgery, Università Cattolica del Sacro Cuore, Roma, Italy; 5 Institute of Cell Biology and Neurobiology, National Research Council of Italy, Monterotondo Scalo, Rome, Italy; 6 Institute of Translational Pharmacology, National Research Council of Italy, Rome, Italy; 7 National Human Neural Stem Cell Resource, Children's Hospital of Orange County Research Institute, Orange, California, United States of America; National Institutes of Health, United States of America

## Abstract

Neural stem cells (NSC) with self-renewal and multipotent properties serve as an ideal cell source for transplantation to treat neurodegenerative insults such as Parkinson's disease. We used Agilent's and Illumina Whole Human Genome Oligonucleotide Microarray to compare the genomic profiles of human embryonic NSC at a single time point in culture, and a multicellular tissue from postmortem adult substantia nigra (SN) which are rich in dopaminergic (DA) neurons. We identified 13525 up-regulated genes in both cell types of which 3737 (27.6%) genes were up-regulated in the hENSC, 4116 (30.4%) genes were up-regulated in the human substantia nigra dopaminergic cells, and 5672 (41.93%) were significantly up-regulated in both cell population. Careful analysis of the data that emerged using DAVID has permitted us to distinguish several genes and pathways that are involved in dopaminergic (DA) differentiation, and to identify the crucial signaling pathways that direct the process of differentiation. The set of genes expressed more highly at hENSC is enriched in molecules known or predicted to be involved in the M phase of the mitotic cell cycle. On the other hand, the genes enriched in SN cells include a different set of functional categories, namely synaptic transmission, central nervous system development, structural constituents of the myelin sheath, the internode region of axons, myelination, cell projection, cell somata, ion transport, and the voltage-gated ion channel complex. Our results were also compared with data from various databases, and between different types of arrays, Agilent versus Illumina. This approach has allowed us to confirm the consistency of our obtained results for a large number of genes that delineate the phenotypical differences of embryonic NSCs, and SN cells.

## Introduction

The adult CNS has a limited capacity to regenerate new neurons, particularly in the substantia nigra, spinal cord, and cortex [1]. In contrast, glial cells can be regenerated in low numbers throughout the adult life span and the numbers generated increase substantially after injury [2]. The embryonic human neural stem cells (NSC) are multipotent cells that have been derived from human embryonic stem cells as well as isolated from fetal tissues [3], [4]. Such cells are viewed as a possible source of neurons for a cell-based therapy of neurodegenerative disorders, such as Parkinson's disease (PD).

In appropriate culture conditions, NSCs can differentiate into specialized cells, including dopaminergic (DA) neurons [5]. A cocktail of growth factors and forskolin increase the conversion of mesencephalic cells [6] to DA neurons. Bcl-Xl and hematopoietic cytokines have been demonstrated to enhance the differentiation of NSCs into DA neurons [7], [8]. Lee et al. [9] established the five-stage differentiation method to induce the embryonic stem cells (ESCs) into DA neurons. Transcription factors like Nurr1 [5] and Pitx3 [10] were introduced into stem cells to enhance the the proof-of- principle for developing different types of allogenic neurons including personalized DA neurons for replacement therapy in PD.

Genome-wide gene expression profiling can be used, providing a more efficiency of DA neuron production. Moreover, the methods to make human DA neurons from human ESCs (hESCs) were established by co-culturing with PA-6 stromal cells [11], [12]. The recently developed protocol of direct reprogramming of adult human somatic cells such as fibroblasts into induced pluripotent stem cells (iPSCs) [13] provided comprehensive molecular understanding of neural differentiation into DA. In-depth examinations of gene expression profiles of embryonic NSC and differentiated DA are likely to reveal information about the “stemness” as well as the pathways involved in DA differentiation.

These relevant genes, once identified, are good candidates to investigate for their role in DA differentiation. In addition, such data are crucial for application to recently reported direct reprogramming protocols, with an overall aim of directly transforming somatic and neural stem cells into more differentiated DA that could be used in cell-replacement therapies.

Whole genome analysis for the process of dopaminergic differentiation from human embryonic NSC has not been reported. To assess the properties of embryonic NSC, and to provide insight about the process of DA differentiation, we employed a whole genome analysis of embryonic NSC, and total RNA isolated from adult human substantia nigra which is rich in dopaminergic cells using Agilent and Illumina Whole Human Gene Expression Microarray (http://www.genomics.agilent.com, http://www.illumina.com ). These microarrays are based on updated transcriptome databases for mRNA targets, and also include probes for lincRNAs (long intergenic non-coding RNAs). With the combination of mRNA and lincRNAs, it is now possible to perform two experiments on a single microarray, confidently predicting lincRNA function. We used this DNA array platform to analyze embryonic NSC populations that were derived from hESC, and DA cell that were isolated from the adult human *substantia* nigra. The results were also compared with data from various databases. This approach has allowed us to confirm the consistency of our obtained results for a large number of genes that delineate the phenotypical differences of embryonic NSCs, and SN cells.

By comparing human embryonic NSC populations and SN cells in the human *substantia* nigra, we identified 13525 genes that are differentially expressed between the two cell populations. Approximately 3737 genes were up-regulated in the embryonic NSC, and 4116 genes were up-regulated in SN cells. Comparison with data sets developed by profiling of the two cell populations showed clear differences, and a series of unique NSC and DA markers were identified. Overall, our data suggest that in comparison to hENSC which showed augmentation of genes involved in regulation of cell cycle and mitosis, the SN cells displayed over-expression of genes related to midbrain development, and DA production.

## Materials and Methods

### Human Embryonic Neural Stem Cells in Culture

Two cryopreserved human embryonic neural stem cells were obtained from Invitrogen (Carlsbad, CA USA) as a commercially available product (N7800-200). The cells were derived from NIH approved H9 (WA09) human embryonic stem cells (hESCs). Three more hENSCs samples were obtained from Dr. Philip Schwartz at National Human Neural Stem Cell Resource, Children's Hospital of Orange County Research Institute, CA (NIH grant HD059967). The cells were plated in a 6-well culture plate coated with polyethyleneimine, and incubated at 37°C in a 5% CO2/95% air incubator in serum-free DMEM/F-12 medium (Invitrogen, Carlsbad, CA, USA) supplemented with a mixture of insulin–transferrin–selenium (ITS) (Invitrogen, Carlsbad, CA, USA), 20 ng/ml recombinant human EGF (Invitrogen, Carlsbad, CA), 20 ng/ml recombinant human bFGF (Invitrogen, Carlsband, CA), and 10 ng/ml recombinant human LIF (Invitrogen, Carlsband, CA), according to the methods described previously [14]. Half of the medium was renewed every 4 days. Following incubation for several months, the embryonic NSC in culture continued to proliferate by forming free floating or loosely attached growing spheres.

For microarray analysis, non-passage embryonic NSC spheres were harvested, replated in a non-coated 6-well culture plate, and incubated further for 72 h in the NSC medium with or without inclusion of 10% fetal bovine serum (FBS) (Invitrogen, Carlsbad, CA, USA).

### Gene expression Profiling and Microarray Analysis

#### RNA Sample Preparation

Total cellular RNA was isolated from embryonic NSC (n = 5) using Trizol (Invitrogen). The RNA quantity was analyzed using the Nano Drop ND1000 (SOP N° TAL009) and the RNA quality is checked using the Bioanalyzer 2100 (Agilent). Total RNA derived from adult human *substantia* nigra (n = 5) was purchased from Clontech (Palo Alto, CA, http://www.clontech.com).

Sample amplification was performed with 200 ng of total RNA using Agilent's Quick Amp Labeling Kit OnoColor to generate complementary RNA (cRNA) for oligo microarrays. cRNA was processed for microarray analysis on a Whole Human Genome Oligonucleotide Microarray (G4112A, 41,000 genes; Agilent Technologies, Santa Clara, CA, USA, and Illumina, USA), according to the manufacturer's instructions.

### Microarray Hybridization

The arrays were hybridized at 60°C for 17 h with the Tecan HS Pro hybridization station in the hybridization buffer containing fluorescence-labeled cRNA.

The microarrays were washed once with 63 SSPE buffer containing 0.005% N-laurylsarcosine for 1 min at room temperature followed by a second wash with pre-heated 0.06× SSPE buffer (37°C) containing 0.005% N-laurylsarcosine for 1 min. The last washing stage was performed with acetonitrile for 30 sec.

### Image and Data Extraction

Fluorescence signals of the hybridized microarrays were detected using Agilent's and Illumina DNA microarray scanner (Agilent and Illumina Technologies) with a resolution of 5 lM.

Agilent Feature Extraction Software (FES) was used to read out and process the microarray image files. The software determines feature intensities and normalized ratios by linear LOWESS with background subtraction, rejects outliers and calculates statistical confidences (P-values). A quality control was performed at this stage. Genes with a P-value smaller than 0.001 were considered significant. Only genes differentially expressed in the ten experiments were considered as relevant genes.

### MIAME

Data were submitted to GEO archive (gene expression omnibus) according to MIAME (Minimum Information About a Microarray Experiment) recommendation. A GEO accession number was assigned: GSE25931.

### Data Mining

Data were processed with GeneSpring GX 10.0.2 software (Agilent Technologies). A fold change (FC)>2 and a P value<0.01 resulted in a selection of 13525 mRNA. Among them, 3737 were up-regulated in embryonic NSC and 4116 were up-regulated in SN cells. For data visualization, hierarchical clustering was performed with the Pearson metric and complete linkage method.

Gene ontology was performed in GeneSpring to identify overrepresented GO-classes compared with the human whole genome, i.e: molecular function, biological process and cellular component [15], [16]. Whole Human Genome was used as the reference group. Statistical significance was calculated with a standard hypergeometric distribution corrected by a Benjamini Yekutelli correction for multiple testing which takes into account the dependency among the GO categories. The minimal length of considered GO-paths was 2. Significance was set at corrected P-value <0.05.

Pathway analysis was used to find direct relationships between entities of interest. This was performed in GeneSpring with the “simple and direct interaction” algorithm. The information was obtained from the literature using Natural Language Processing algorithms to search PubMed abstracts.

Hierarchical clustering of these differentially expressed genes was conducted using the software, Cluster 3.0, with centroid linkage and visualized using Tree View. (http://bonsai.ims.utokyo.ac.jp/mdehoon/software/cluster/software.htm). Principle component analysis (PCA) was conducted using the bioconductor packages (www.bioconductor.org).

### Functional Annotation and Molecular Network Analysis

Raw and processed gene expression data have been deposited in NCBI's Gene Expression Omnibus (GEO, http://www.ncbi.nlm.nih.gov/geo/, GEO Series accession number GSE25931). Whole human genome arrays from human embryonic NSC, and SN cells underwent weighted averaging by Rosetta Resolver software to produce an average differential expression ratio for every gene. Although there are cases on the array where more than one probe is used to query different parts along the sequence of a single gene, and in some instances, identical probes to the same gene are replicated mainly for QC purposes, the vast majority of genes are queried using a single probe with the Agilent and Illumina platform. Functional annotation of significant genes identified by microarray analysis was searched by the web-accessible program named Database for Annotation, Visualization and Integrated Discovery (DAVID) version 2009, from the National Institute of Allergy and Infectious Diseases (NIAID (NIH) (david.abcc.ncifcrf.gov) [17], [18]. DAVID covers more than 40 annotation categories, including Gene Ontology (GO) terms, protein–protein interactions, protein functional domains, disease associations, biological pathways, sequence general features, homologies, gene functional summaries, and tissue expressions. By importing the list of the National Center for Biotechnology Information (NCBI) Entrez Gene IDs, this program creates the functional annotation chart, an annotation-term-focused view that lists annotation terms and their associated genes under study. To avoid excessive count of duplicated genes, the Fisher's exact test is calculated based on corresponding DAVID gene IDs by which all redundancies in original IDs are removed. Gene ontology (GO) and KEGG molecular pathway analysis was performed to identify possible enrichment of genes with specific biological themes using both the data set as a whole and then in the individual K-means clusters. DAVID calculates a modified Fishers Exact p-value to demonstrate GO or molecular pathway enrichment, where p-values less than 0.05 after Benjamini multiple test correction are considered to be strongly enriched in the annotation category.

### Correlation with other Gene Expression Data from Various Databases

Additional expression data sets in form of CEL-files were obtained from the web (http://www.ncbi.nlm.nih.gov/geo/) or (http://wombat.gnf.org/suppl.html#reqdata_geneatlas). Datasets included the NCI60-dataset (http://wombat.gnf.org/samples/NCI60_sample_info.htm), the GNF-SymAtlas [19] and the “breadth-of-expression” dataset [20]. Own experimental data and external data were put together and low-level-corrected using the RMA-algorithm [21]. Expression data were then exported in log2-scale. In addition, individual expression profiles were fitted by means of median polish to a reference profile in regard to intensity distribution and RNA quality. Over-expressed genes were determined to exceed a minimal difference of 2.5 (in log2-scale) between their individual intensity and the median intensity of all analyzed chips. Only genes over-expressed in all chips for each respective cell type were used for analysis and comparison with other data. To detect relevant statistical differences/similarities within the main cluster of neural tissues, we extracted the expression profiles derived from all available neural tissues for further statistical analyses, while keeping the changes introduced by the global normalization procedure.

### Pathway Analysis

762 pathways (BioPax format pathways with .owl extension) were imported into GeneSpring GX from reactome database (reactome.org) to perform “find significant pathways” tool. This tool allows to investigate if there is a significant enrichment of my genes of interest in a particular pathway. The p-value is calculated using the hypergeometric distribution.

## Results

### Obtaining and Characterizing Samples

Ten cell samples were selected and subdivided into two groups. The first group was composed of five samples of hESC. As representatives of dopaminergic neurons, we had included five RNA samples collected from normal human brain (substantia nigra) that are known to be rich in dopaminergic neurons. The total RNA samples of adult human substantia nigra for the first two samples were pooled from 19 male/female Caucasians, ages: 25–64 whose cause of death was given as “sudden death.” The last three samples of human SN were collected on an individual base from male Caucasians, ages: 25, 53, and 67.

**Figure 1:**
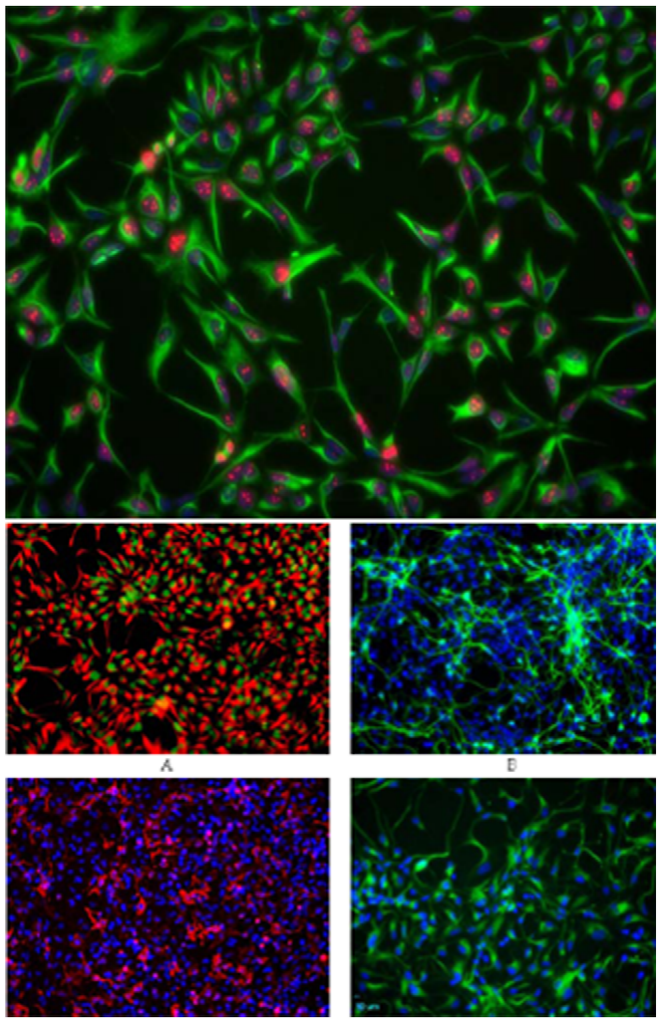
Fluorescence image (20×) of GIBCOR hNSCs at passage 3 that have been cultured in StemProR NSC SFM and stained for the NSC phenotype markers nestin (green) and the proliferation marker Ki67 (red, a). Cell nuclei were counterstained with DAPI (blue,a). Approximately 90% of the cells stain positive for the undifferentiated NSC marker nestin and the proliferation marker Ki67. Lack of Oct4 staining indicates that there are no remnant hESCs in the culture (data not shown) (Invitrogen, Manual part no. A11592, MAN0001758). Fluorescence images (20×) of GIBCOR hNSCs that have been cultured in StemProR NSC SFM for three passages, and then allowed to differentiate into neurons, oligodendrocytes, or astrocytes. Upon directed differentiation, cells start to lose the undifferentiated NSC marker, nestin, but stain positive for the differentiated cell type markers Dcx, GalC, and GFAP. Cells were stained for the undifferentiated NSC markers nestin (red, b) and SOX2 (green, c) prior to directed differentiation. Cell were then differentiated into neurons and glial cells, and respectively stained for the neuronal marker Dcx (green, c), for the oligodendrocyte marker GalC (red, d), or for the astrocyte marker, GFAP (green, e). The nuclei were counterstained with DAPI (blue) in panels B–D (Invitrogen, Manual part no. A11592, MAN0001758).

Based on previous characterization by Invitrogen, the human neural stem cells used in the present study were self-renewing, multipotent stem cells of the nervous system derived from the NIH approved H9 (WA09) human embryonic stem cells (hESCs). The cells retain their normal female human karyotype and their potential to differentiate into neurons and glial cells after multiple passages. They stain positive for the neural stem cell-type specific markers nestin and SOX2, the proliferation marker Ki67 (>80%), and for embryonic stem cell-specific marker Oct4 (≤5%) (Fig. 1).

The hNSCs spontaneously differentiate into neurons, oligodendrocytes, or astrocytes upon withdrawal of bFGF and EGF from culture media. Alternatively, they can be enriched toward a specific lineage upon selection on differentiation medium.

Based on previous characterization by Clontech, the total RNA from human substantia nigra was analyzed by capillary electrophoresis (CE) using an Agilent 2100 Bioanalyzer. RNA concentration and purity were evaluated by UV spectrophotometry. Both the area ratio of the 28S/18S rRNA peaks, and the proportion (relative percentage) of these two peak areas to the total area under the electropherogram provide reliable quantitative estimates of RNA integrity.

### Data Acquisition Analysis and Verification

We used Whole Human Genome Oligonucleotide Microarray (G4112A, 41,000 genes; Agilent and Illumina Technologies, USA) to monitor gene expression in human embryonic NSC, and human SN cells which are known to be rich in dopaminegic neurons.

The Whole Human Genome Oligo microarray is comprised of approximately 41,000 (60-mer) oligonucleotide probes, which span conserved exons across the transcripts of the targeted full-length genes. These probes represent the human genome, full-length and partial human genes from a number of major public sources. The sequence and annotation information used in this microarray product is available through Agilent, Illumina and publicly-available databases such as RefSeq, GoldenPath, Ensembl and more.

### Correlation, Cluster Analysis and Comparison of hENSC with Human SN Cells

**Figure 2:**
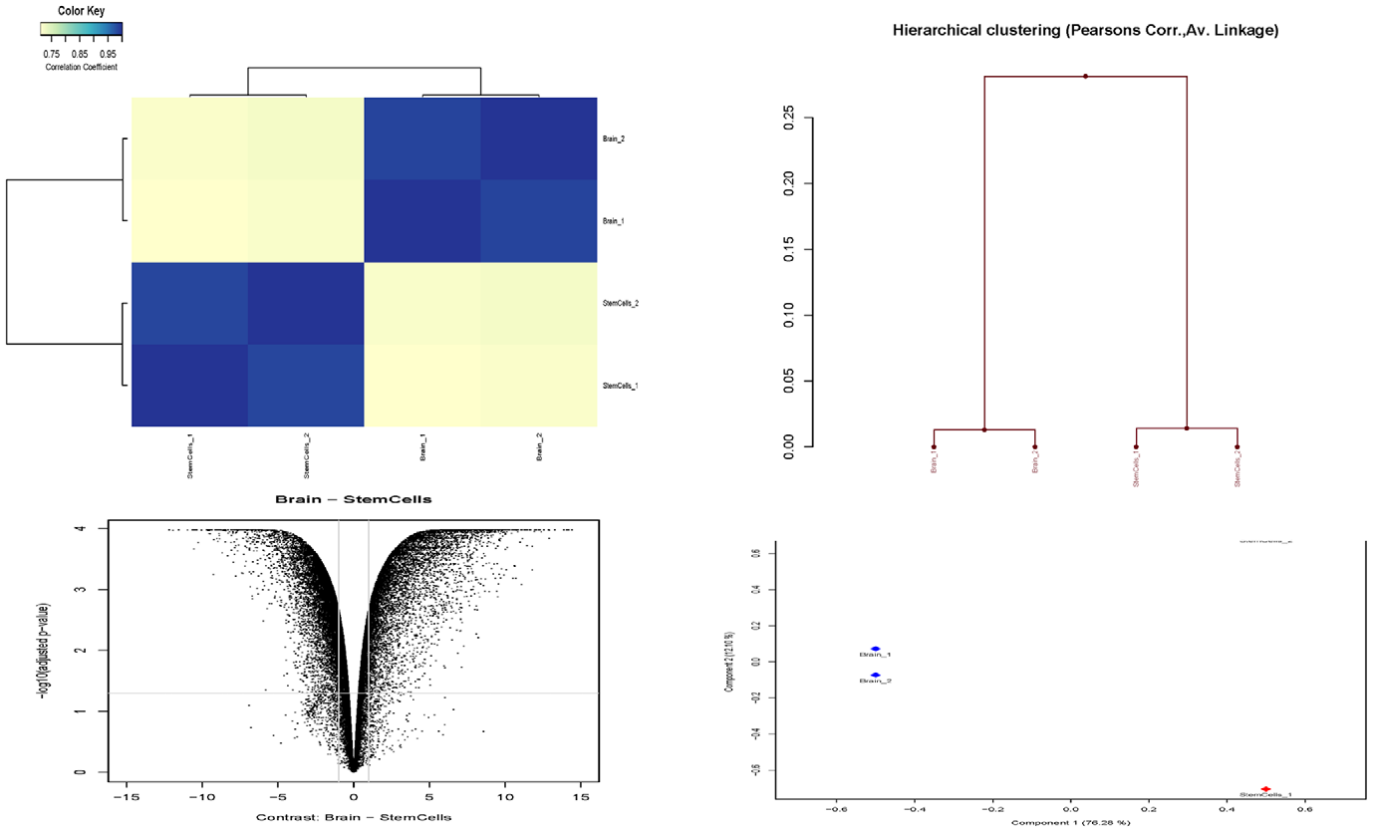
The correlation between human embryonic neural stem cells (StemCells_1,2) and dopaminergic cells (Brain_1,2), and also among the two arrays of the same sample. There was no correlation (yellow colors) between hENSC and DA cells samples. But there was a strong correlation (blue colors) between the arrays from the same samples (A). Hierarchical clustering analysis using GeneSpring GX Software. Hierarchical clustering dendrogram of relative gene expression in different populations was generated using the GeneSpring GX Software (B). hENSCs (stem_1, and stem_2) clustered together and are distinguished from human substantia nigra dopaminergic cells (Brain_1, and Brain_2). Unsupervised hierarchical clustering based on overall gene expression profiles reveals two distinct clusters corresponding to the two cell groups. This suggests that the samples from the two cell types (hENSC vs DA cells) do indeed represent two statistically distinct populations suitable for valid comparison. Scatter-plot used to quickly identify discrepancies in gene expression profiles between hENSC and DA cells from human *substantia nigra* (C). Both cell populations could be clearly discriminated based of differences in their gene expression profiling. Principal component analysis (PCA) which is useful when we expect samples to be linearly (or even monotonically) related to each other (D). As you see here samples from the hENSC and from human *subastantia nigra* DA cells are completely different.

Heatmap correlation, hierarchical clustering analysis, principal component analysis (PCA), and scatter-plot were conducted using GeneSpring GX Software. Heatmap correlation (Figure 2a), a dendrogram analysis (Figure 2b), scatter-plot (Figure 2c, and PCA (Figure 2d) illustrate that hENSC samples clustered together and could be discriminated from human SN cells analyzed in this study. From all these data, we concluded that the overall gene expression profile seen in hENSC could be discerned from that seen in human substantia nigra dopaminergic cells.

RNA extracted from human embryonic NSC and adult human substantia nigra was used to produce cDNA and hybridized to Agilent's and Illumina Whole Human Genome microarray. Genes expressed differentially between hENSC and SN cells were identified using two criteria: those with a greater than 2-fold difference in normalized expression between the two cell populations, and those that were flagged “present” at one type and “absent” at the other.

We considered only genes that were regulated at least two-fold in a significant manner based on replicate hybridizations (p<0.05). Using these criteria, we identified 1830 up-regulated and 1932 down-regulated mRNA in DA SN *vs* hE_NSC.

Of these, the top ten most differentially expressed probe sets determined using the two approaches are shown in Table S1 for hENSC and for SN cell. Genes in all tables were categorized, annotated and their GO analysis was carried out based on functional information obtained in online databases.

We found that the set of genes expressed more highly in hENSC is enriched in molecules known or predicted to be involved in GO:0000166: nucleotide binding, GO:0003723: RNA binding, GO:0005515: protein binding, GO:0005634: nucleus, GO:0005737: cytoplasm, GO:0017148: negative regulation of translation, GO:0042035: regulation of cytokine biosynthetic process, GO:0045182: translation regulator activity, GO:0048027: mRNA 5′-UTR binding, GO:0000087: M phase of mitotic cell cycle (Table 1, and Table S1). This suggests that at hENSC are in a state of active metabolism and growth, and some of them may potentially still be undergoing proliferation. The higher expression level of ribosomal genes is also indicative of cells in a state of growth.

**Table 1:** Functional Annotation Clustering using DAVID for the top 1200 unregulated genes (Log2≤5–12) of hENSC.

Annotation Cluster 1	Enrichment Score 27.56	Count	P_Value	Benjamini
GOTERM_BP_FAT	M Phase	63	2.2E-47	3.9E-44
GOTERM_BP_FAT	cell cycle	87	2.5E-47	2.2E-44
GOTERM_BP_FAT	cell cycle phase	67	1.1E-45	6.5E-43
GOTERM_BP_FAT	cell cycle process	74	3.1E-44	1.4E-41
SP_PIR_KEWORDS	mitosis	47	2.4E-42	8.0E-40
GOTERM_BP_FAT	mitosis	50	6.2E-41	2.2E-38
GOTERM_BP_FAT	nuclear division	50	6.2E-41	2.2E-38
SP_PIR_KEWORDS	cell cycle	64	7.1E-41	1.2E-38

One hundred ten annotation clusters were obtained. The annotation cluster one included M phase, cell cycle, cell cycle phase, cell cycle process, mitosis with an enrichment score of 27.56. On the very right you find the Benjamini corrected enrichment score; the lower the better. The functional annotation tool now allows several methods for adjusting p-values for multiple testing. The Benjamini–Hochberg–Yekutieli procedure controls the false discovery rate under dependence assumptions.

On the other hand, the genes enriched in SN samples include a different set of functional categories, namely GO:0007268: synaptic transmission, GO:0007417: central nervous system development, GO:0019911: structural constituent of myelin sheath, GO:0033269: internode region of axon, GO:0042552: myelination, GO:0042995: cell projection, GO:0043025: cell somata, GO:0043209: myelin sheath, GO:0005783: endoplasmic reticulum, GO:0005789: endoplasmic reticulum membrane, GO:0005792: microsome, GO:0006633: fatty acid biosynthetic process, GO:0006665: sphingolipid metabolic process, GO:0006810: transport, extracellular region, GO:0006811: ion transport, GO:0031643: positive regulation of myelination, GO:0001518: voltage-gated sodium channel complex, GO:0005244: voltage-gated ion channel activity, GO:0005248: voltage-gated sodium channel activity, GO:0006811: ion transport, GO:0006814: sodium ion transport, GO:0007268: synaptic transmission. (Table S1, and Table S2). This result suggests that a key role of DA in the adult human *substantia* nigra is to support/modulate synaptic transmission, central nervous system development, myelin sheath formation, and voltage-gated ion channel activity where we identified some potential molecular players that may mediate this role.

### An overview of Human Embryonic Neural Stem Cells Transcriptome

We started our DNA microarray data analysis by studying expression level of the generally accepted stem cell markers such as POU, transcription factor 1 (Oct3/4), teratocarcinoma-derived growth factor (Tdgf1), Enk-pending (Nanog), undifferentiated embryonic cell transcription factor 1 (Utf1), and DNA methyltransferase 3B (Dnmt3b) in samples of hENSCs. The POU domain, and DNA methyltransferase 3B (Dnmt3b) were significantly increased to 2.80, and 4.44 fold respectively. Class 5, transcription factor 1 (Oct3/4), signal transducer and activator of transcription 3 (Stat3), teratocarcinoma-derived growth factor (Tdgf1), Enk-pending (Nanog), undifferentiated embryonic cell transcription factor 1 (Utf1) were not significantly increased. These results might indicate the absence of any undifferentiated ESC within our hENSC samples used.

To further test for the presence of undifferentiated embryonic stem cell markers, samples from hENSC were assayed for the presence of the endodermal gene AFP and the mesodermal gene Eomes. The AFP, and Eomes were not significantly increased in hENSC samples. Eomes was increased by 2.82 fold in our human substantia nigra cells which is quite logical, taking into consideration the mesodermal origin of some types of glial (microglia) cells, which are known to co-exist with DA cells. The differentiation status of hENSC was also checked by the expression level of the lineage markers of three layers: T (brachyury) for mesoderm, Nodal for ectoderm, and Gata4 (GATA binding protein 4) for endoderm. None of them were significantly increased in our examined samples of ENSC.

Next, we studied markers of NSC such as Nestin, Sox1, Sox2, CD133, and Musashi1. Nestin, Sox1, Sox2, Musashi1 were increased 3.19, 2.58, 2.23, 1.49 fold respectively, while CD133 was not significantly increased.

Of the 17 SOX-family transcription factors present on the array, only SOX1, 11, 12, and 13, which exhibit neural tube/progenitor expression or function, were detected and increased 4.66, 5.71, 2.58, and 3.04 fold respectively.

Next, we tested the undifferentiated nature of hENSC by examining the expression profile of more mature neuronal markers such as choline acetyltransferase, tyrosine hydroxylase (TH) or those of late glial markers such as glial fibrillary acidic protein (GFAP), myelin associated glycoprotein (MAG), and oligodendrocyte transmembrane (OSP). We did not detect expression of any of the more mature neuronal and glial markers in the hENSC samples, suggesting that the neural cells in the sample were indeed undifferentiated NSC.

Next, we chose a set of regulated genes that are associated with NSC proliferation or differentiation. Cystatin C acts as a necessary cofactor for FGF-2 in neural stem cells [22]. The expression of cystatin C was increased 3.89 fold in human *substantia* nigra cells but showed no significant increase in hENSC, further indicating the undifferentiated nature of hENSC samples. Synaptotagmin XI belongs to a family of proteins involved in synaptic vesicle trafficking. The increase in expression of synaptotagmin XI as differentiation proceeded was confirmed where it was increased by 2.5 fold in human substantia nigra cells but showed no significant increase in hENSC. Myelin proteolipid protein, PLP, is an integral membrane protein that accounts for 50% of the protein mass in the myelin sheath. The expression of PLP1, and PLP2 was stimulated by 6.71, and 3.88 fold respectively in human *substantia* nigra cells, but were not detected in hENSC samples.

In contrast, no significance up-regulation of genes related to various extracellular matrix proteins, such as various collagen isoform and other genes related to connective tissue and its metabolism (LOXL1, LOXL2, and LUM) was encountered. Also, genes related to fully differentiated neural tissue like the hippocampus expressed neuronal and glial genes like NTRK2, NTRK3, MBP, and GFAP were not demonstrated.

The genomic profiling of our hENSC line was characterized by a lower expression of MHCII genes and myelin components such as peripheral myelin protein two (PMP2), myelin-associated glycoprotein (MAG), or the myelin-oligodendrocyte glycoprotein (MOG). However, PMP2, PMP22 and MOG were up-regulated to 10.17, 4.43, and 9.70 fold respectively in SN cells. Interestingly, this study revealed that CD44 and GDF1/LASS1 were not expressed in hENSCs line. Our data revealed that genes specific for differentiated neurons such as SNAP25 and neurogranin (NRGN) were markedly up-regulated to 7.49 fold in SN cells, while it was not detected in hENSCs. In the present study, genes specifically expressed in adult NPCs such as low-affinity nerve growth factor receptor precursor (p75, NGFR), Nestin (NES), the dual specific phosphatase 10 (DUSP10), the CCmotif containing chemokine receptor seven (CCR7), the transcription factor four (TCF4), the G protein-coupled receptor 17 (GPR17), and the chondroitin sulfate proteoglycan four (CSPG4) were specifically under-expressed in hENSCs line.

### An overview of Human Subastantia nigra Dopaminergic Cells Transcriptome

The substantia nigra sample used in the present study is a total mRNA extracted from dissected human SN, and not from DA neurons isolated from such tissue so it is rich in DA, and glial cells with nearly an entire absence of NSC. First, we checked the expression of neuronal and glial markers. Tubulin beta 2A and tubulin beta 4 were increased by 2.26, and 5.66 fold respectively. The glial fibrillary acidic protein (GFAP) gene was increased by 14.18 fold. Neurogenic differentiation 1 (NeuroD1), is a transcription factor that indicated the commencement of neural differentiation: It was significantly increased by 3.43 fold.

The genes related to midbrain development, such as Nr4a2 (nuclear receptor subfamily 4, group A, member 2, Nurr1) and En1 (engrailed 1) were increased 3.76, and 6.41 fold respectively. In addition, the transcriptions of the genes such as Ddc (dopamine decarboxylase, AADC), Slc6a3 (solute carrier family 6, member 3, DAT), and Th (tyrosine hydroxylase) were significantly increased to 8.08, 4.01, and 5.91 respectively.

We also scrutinized gene expression in human substantia nigra and found many transcripts that were unregulated in them. Many of these genes have known or presumed function during neural development and differentiation, including FGF signaling (FGF3, FGFR2, FGFR12) which were increased by 2.16, 3.66, 3.71 fold respectively. WNT signaling (FZD3) was not significantly increased in human substantia nigra. Neurogenic functions (DLK1, VEGF) were increased 3.20, 2.37 fold respectively, and neurotrophin signaling (NTRK2) was increased to 3.40 fold. Common markers of neuronal cell function were also up-regulated such as neurofilaments NEFL, MAP2, and NCAM1 which were increased by 4.30, 2.10, 14.16 fold respectively. Consistent with this, the midbrain dopaminergic marker LMX1B (LIM homeobox transcription factor 1, beta) was increased 2.42 fold.

Several transcription factors that are involved in the specification of the midbrain dopaminergic lineage, EN1, and LMX1B, were all expressed at higher levels in our human substantia nigra, at approximately 6.41, 2.42 fold, respectively.

Interestingly, the GIRK2 channel protein (a marker of A9 dopaminergic neurons) [23], which is the major dopamine neuron subtype depleted in Parkinson's disease [24], [25], [26] was not detected in our human substantia nigra samples. Expression of DβH, a more specific marker for other catecholaminergic neurons, was also not significantly increased. This expression analysis suggested elevated expression of dopaminergic transcription factors and some markers of differentiated neurons in our examined samples of human SN cells.

Next, we studied the expression profiling of more than 28 genes (Table S2) that are modulated in the dopaminergic system.

### Functional Annotation Clustering of hENSC

Functional annotation of significant genes identified by microarray analysis was searched by the web-accessible program named Database for Annotation, Visualization and Integrated Discovery (DAVID) version 2009. Clustering for the top 1200 unregulated genes (Log2 5–12) of hENSC using DAVID, we identified 110 annotation clusters. The annotation cluster 1 (Table 1) showed the highest enrichment score of 27.56 and included genes related to M phase, cell cycle, cell cycle phase, cell cycle process, mitosis.

### Functional Annotation Clustering of Substantia nigra Dopaminergic Cells

Clustering for the top 1200 unregulated genes (Log2 5–14) of substantia nigra samples using DAVID identified 185 Cluster annotation clusters. The annotation cluster 1 (Table 2) showed the highest enrichment score of 13.07 and included genes related to glycoproteins, disulphide bonds, and signals. The annotation cluster group 4 (Table 3) with an enrichment score of 7.58 contains genes related to neuronal cell functions and differentiation such as transmission of nerve impulses, synaptic transmission, cell-cell signaling, and neurological system processes. The transmission of nerve impulse group included genes that regulate the neurological system process by which a signal is transmitted through the nervous system by synaptic transmission and the sequential electrochemical polarization and depolarization that travels across the membrane of a nerve cell (neuron) in response to stimulation.

**Table 2:** Functional Annotation Clustering using DAVID for the top1200 unregulated genes (Log2≤5–12) of human substantia nigra.

Annotation Cluster 1	Enrichment Score 13.07	Count	P_Value	Benjamini
SP_PIR_KEWORDS	glycoprotein	187	9.9E-19	4.7E-16
UP_SEQBP_ FEATURE	glycosylation site:Nlinked	176	3.2E-16	4.5E-13
SP_PIR_KEWORDS	disulfide bond	129	1.6E-12	1.9E-10
SP_PIR_KEWORDS	signal	138	3.2E-12	3.0E-10
SP_PIR_KEWORDS	signal peptide	138	5.1E-12	1.4E-9
UP_SEQBP_ FEATURE	disulfide bond	122	4.8E-11	41E-8

Annotation Cluster 2	Enrichment Score 11.7	Count	P_Value	Benjamini
SP_PIR_KEWORDS	glycoprotein	187	9.9E-19	4.7E-16
UP_SEQBP_ FEATURE	glycosylation site:Nlinked	176	3.2E-16	4.5E-13
SP_PIR_KEWORDS	membrane	231	3.1E-15	7.4E-13
UP_SEQBP_ FEATURE	topological domain:Cytoplasmic	147	8.4E-14	5.7E-11
UP_SEQBP_ FEATURE	transmembrane region	187	1.9E-12	8.8E-10
UP_SEQBP_ FEATURE	topological domain:Extracellular	123	1.9E-12	6.6E-10
SP_PIR_KEWORDS	transmembrane	187	3.5E-12	2.8E-8
GOTERM_CC_FAT	plasma membrane	166	1.7E-10	1.8E-6
GOTERM_CC_FAT	intrinsic to membrane	209	5.2E-8	3.3E-6
GOTERM_CC_FAT	integral to membrane	198	1.1E-6	4.3E-5

185 clusters were obtained. The annotation cluster 1 showed the highest enrichment score of 13.07 and included genes related to glycoproteins, disulphide bond, and signals.

**Table 3:** Functional Annotation Clustering using DAVID for the top 1200 unregulated genes (Log2≤5–12) of human substantia nigra.

Annotation Cluster 4	Enrichment Score 7.58	Count	P_Value	Benjamini
GOTERM_BP_FAT	transmission of nerve impulse	34	1.5E-10	3.3E-7
GOTERM_BP_FAT	synaptic transmission	30	9.7E-10	1.1E-6
GOTERM_BP_FAT	cell-cell signaling	43	4.7E-9	3.5E-6
GOTERM_BP_FAT	neurological system process	51	6.9E-4	4.3E-2

Annotation Cluster 5	Enrichment Score 7.3	Count	P_Value	Benjamini
SP_PIR_KEWORDS	cell adhesion	32	9.9E-19	2.1E-6
UP_SEQBP_ FEATURE	cell adhesion	44	3.2E-16	7.5E-5
SP_PIR_KEWORDS	Biological adhesion	44	3.1E-15	6.1E-5

Annotation Cluster 6	Enrichment Score 5.94	Count	P_Value	Benjamini
UP_SEQBP_ FEATURE	signal	138	1.9E-12	3.2E-12
UP_SEQBP_ FEATURE	signal peptide	138	1.9E-12	1.4E-9
SP_PIR_KEWORDS	secreted	65	3.5E-12	4.3E-3
GOTERM_CC_FAT	extracellular region part	48	1.7E-10	3.8E-3
GOTERM_CC_FAT	extracellular space	37	5.2E-8	5.4E-3
GOTERM_CC_FAT	extracellular region	75	1.1E-6	1.0E-1

Annotation Cluster 7	Enrichment Score 5.21	Count	P_Value	Benjamini
GOTERM_CC_FAT	response to wounding	35	1.1E-6	3.6E-4
GOTERM_CC_FAT	inflammatory response	26	1.3E-6	3.6E-4

185 annotation clusters were obtained. The annotation cluster group 4 with an enrichment score of 7.58 contains genes related to nervous system and neurons such as transmission of nerve impulse, synaptic transmission, cell-cell signaling, and neurological system process.

The annotation cluster group 50 with an enrichment score of 1.36 contains genes specific to dopaminergic neurons such as GABA receptor activity (5 genes), postsynaptic cell membrane (20 genes), neurotransmitter binding (9 genes), ligand-gated ion channel activity (10 genes), GABA-A receptor activity (4 genes), gamma-aminobutyric acid signaling pathway (4 genes), and Gamma-aminobutyric acid A receptor (4 genes).

### Gene ontology (GO) of hE_NSC and human Substantia nigra Dopaminergic Cells

Gene ontology (GO) and KEGG molecular pathway analysis was performed to identify possible enrichment of genes with specific biological themes using both the data set as a whole and then in the individual K-means clusters. DAVID calculates a modified Fishers Exact p-value to demonstrate GO or molecular pathway enrichment, where p-values less than 0.05 after Benjamini multiple test correction are considered to be strongly enriched in the annotation category. GO clustering using DAVID for the top 1200 unregulated genes (Log2≤5–12) of hENSC. Forty clusters were obtained that include ATP binding (62 genes), adenyl ribonucleotide binding (62 genes), adenyl nucleotide binding (63 genes), purine nucleoside binding (63 genes), nucleoside binding (63 genes), ribonucleotide binding (63 genes), purine ribonucleotide binding (63 genes), purine nucleotide binding (64 genes), nucleotide binding (71 genes) and others.

Gene ontology (GO) and KEGG molecular pathway analysis was performed to identify possible enrichment of genes for human substantia nigra dopaminergic cells.

GO clustering using DAVID for the top1200 unregulated genes (Log2≤5–12) of dopaminergic cells. Ninety one clusters were obtained that include channel activity (31 genes), passive transmembrane transporter activity (31 genes), substrate specific channel activity (29 genes), gated channel activity (25 genes), GABA receptor activity (5 genes).

### KEGG Pathway Analysis of hE_NSC and Human Substantia Nigra

**Figure 3:**
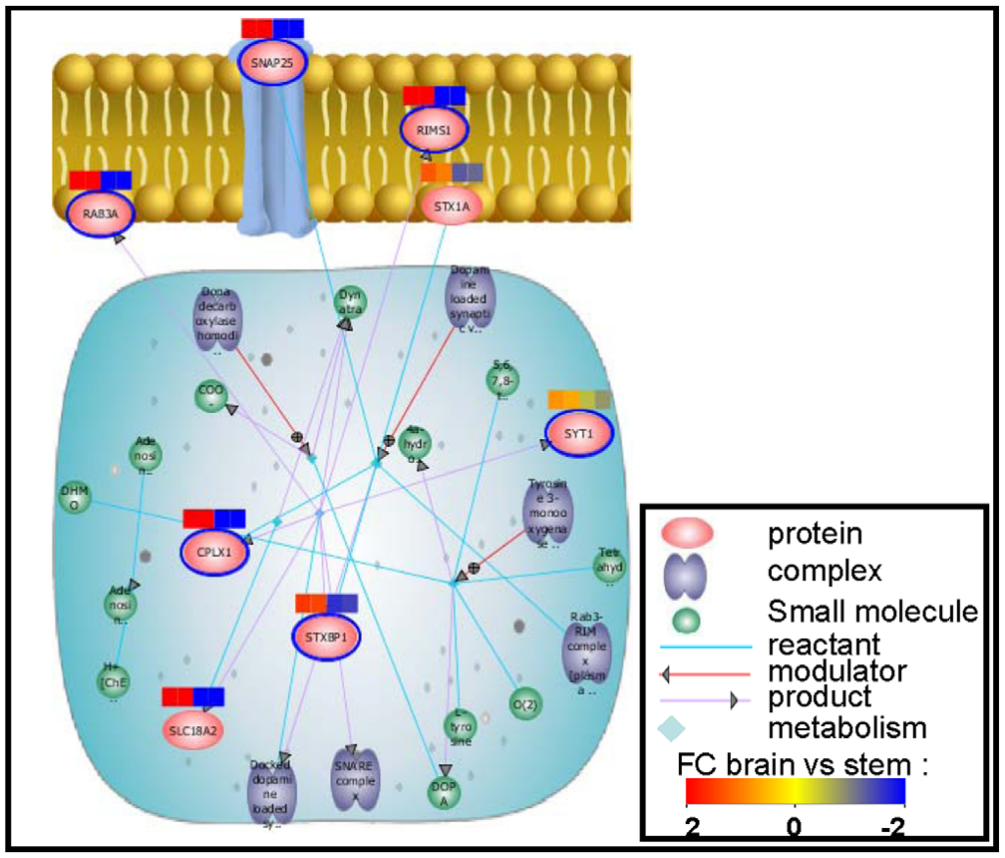
Dopamine neurotransmitter release cycle pathway. The 1830 up-regulated mRNA set are significantly enriched by mRNA coding for proteins involved in “dopamine neurotransmitter release cycle” pathway. The network was generated by “find significant pathway” function in GeneSpring GX v11 (p<0.05). Connections were based on known interactions between these proteins within the Reactome database. The biological relationship between two proteins is represented as a line (an edge). The proteins with a blue halo belong to the 1830 mRNA up-regulated set in brain *vs* stem cells. Red and blue colored squares indicate genes with up- and down-regulated expression, respectively. The intensity of the colors highlights the fold change (FC) in brain *vs* stem cells samples. The nature of the relationships between the proteins are shown in legend. SNAP25, synaptosomal-assiociated protein 25; RIMS1, regulating synaptic membrane 1; RAB3A, RAB3A interacting protein; SYT1, synaptotagmin; CPLX1, complexin 1; STXBP1, syntaxin binding protein 1.

**Figure 4:**
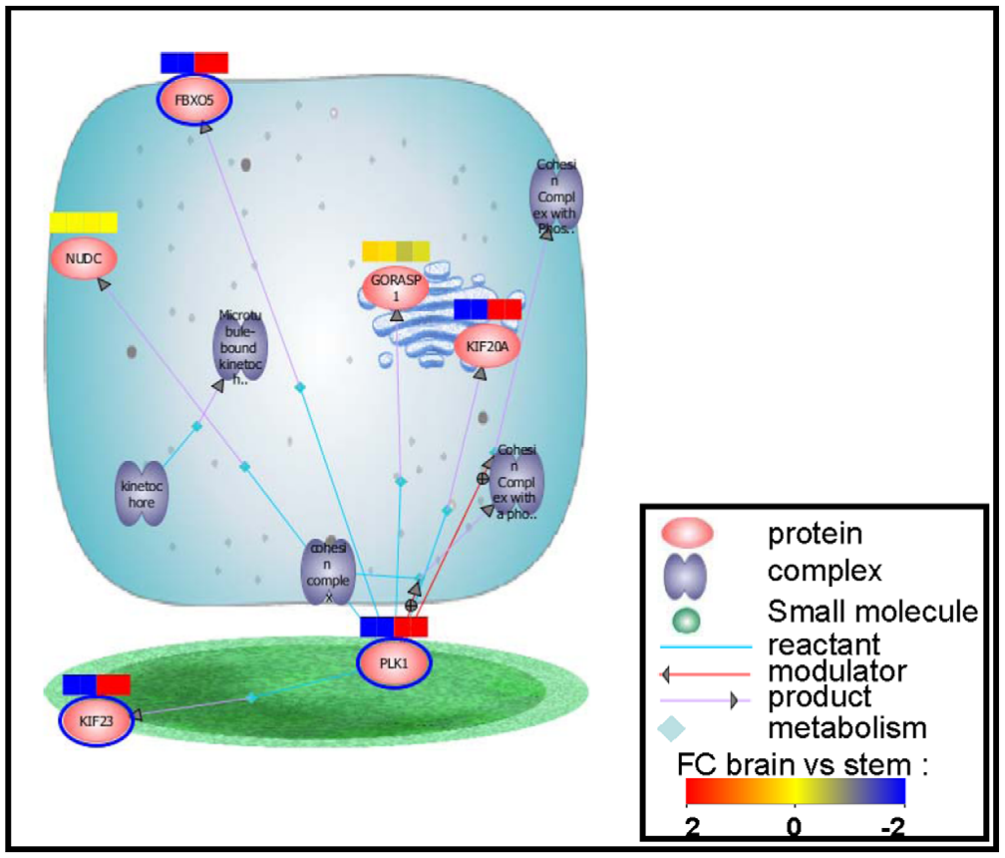
M phase Pathway. Ninety-one pathways were significantly found in the 1932 down-regulated mRNA set, among them we noted “M phase” The 1932 down-regulated mRNA set are significantly enriched by mRNA coding for proteins involved in “M phase” pathway.

The 1830 up-regulated and 1932 down-regulated mRNA in SN vs hE_NSC were submitted to “find significant pathways” tool into GeneSpring GX. Twenty-one pathways were significantly found in the 1830 up-regulated mRNA set, among them we noted “dopamine neurotransmitter release cycle” (Figure 3). Ninety-one pathways were significantly found in the 1932 down-regulated mRNA set, among them we noted “M phase” (Figure 4).

KEGG pathway analysis of the top 1200 up-regulated genes (Log2≤5–12) of hENSC showed significant enrichment for eight molecular pathways: cell cycle, oocyte meiosis, p53 signaling pathway, progesterone-mediated oocyte maturation, viral myocarditis, ABC transporters, vibrio cholerae infection, and homologous recombination.

The cell cycle pathway contained 22 genes representing 6.6% of the genes detected by KEGG pathway analysis. The cell cycle pathway is dominated by genes regulating cell proliferation and mitosis such as E2F transcription factor 2, TTK protein kinase, c-abl oncogene 1, receptor tyrosine kinase, cell division cycle 2, G1 to S and G2 to M, cyclin A2, cyclin B1, cyclin B2, cyclin-dependent kinase 2, pituitary tumor-transforming 1; pituitary tumor-transforming 2, protein kinase CHK2-like; and tumor suppressor protein p53.

The enriched molecular pathways and GO categories identified for hENSC were predominantly driven by genes in the cluster up-regulated with dramatic changes ranging from 5–12 fold increases in expression. In addition to genes involved in cell proliferation and cell division regulation such as TTK protein kinase, c-abl oncogene 1, receptor tyrosine kinase, cell division cycle 2, G1 to S and G2 to M, cyclin A2, cyclin B1, cyclin B2, cyclin-dependent kinase 2, protein kinase CHK2-like, several genes related to tumor transformation and tumor control were also encountered such as pituitary tumor-transforming 1 and pituitary tumor-transforming 2 and tumor suppressor protein p53. The differential expression of these genes is probably result of the embryonic nature of our examined cell lineage.

KEGG pathway analysis of the top1200 unregulated genes (Log2≤5–12) of SN samples showed significant enrichment for 24 molecular pathways: cell adhesion molecules (CAMs), (16 genes), neuroactive ligand-receptor interaction (17 genes), tyrosine metabolism (6 genes), beta-Alanine metabolism (4 genes), tryptophan metabolism (5 genes), and calcium signaling pathway (11 genes). The cell adhesion molecules (CAMs) pathway contained 16 genes representing 1.3% of the genes detected by KEGG pathway analysis. The cell adhesion molecules (CAMs) pathway is dominated by genes in the cluster up-regulated with dramatic increases ranging from 5–12 fold. The genes are involved in regulation of neuronal pre-and postsynaptic activities as well as Schwann cells and oligodendrocyte differentiation such as neural cell adhesion molecule 1, contactin 1, and contactin 2 (axonal), and claudin 11.

We next examined the top 15 up-regulated genes in both human embryonic NSC, and SN samples. In hENSC the most up-regulated, gene showing a 12.17 fold increase was insulin-like growth factor 2 mRNA binding protein 1. This gene encodes a member of the insulin-like growth factor 2 mRNA-binding protein family. The protein encoded by this gene contains four K homology domains and two RNA recognition motifs. It functions by binding to the mRNAs of certain genes, including insulin-like growth factor 2, beta-actin and beta-transducin repeat-containing protein, and regulating their translation.

The over expression of GFAP might indicate the presence of some glial cell contamination within our examined human SN samples. In addition, the significant 12.57 fold increase in apolipoprotein D might indicate susceptibility for Alzheimer's disease in our examined human SN cells.

One focus of our study was to characterize a complex series of signaling programs potentially involved in the priming process of hNSCs. A better understanding of these signaling pathways at a cellular level would be of great assistance to the elucidation of the mechanisms underlying stem cell lineage specification and to the discovery of potential targets for stem cell therapy, gene therapy and pharmaceuticals. The signaling molecules changed during differentiation of hNSC into SN samples were including Wnt, and Notch pathways.

Notch signaling pathway has been reported widely to regulate cell fate determination, cell polarity and differentiation. Notch pathway components were regulated differently. For example, the transcription factor enhancer of split and the ligand deltex, and TACE were down regulated while CBF1 interacting corepressor were up-regulated to 2.02 fold in our hENSC samples.

## Discussion

Only a limited number of genes are currently recognized as established stem cell markers such as POU domain, class 5, transcription factor 1 (Oct3/4), signal transducer and activator of transcription 3 (Stat3), teratocarcinoma-derived growth factor (Tdgf1), Enk-pending (Nanog), undifferentiated embryonic cell transcription factor 1 (Utf1) and DNA methyltransferase 3B (Dnmt3b) [27]. In the present study, genomic profiling of the embryonic human NSCs showed significant over expression of POU (2.80 fold), and DNA methyltransferase 3B (Dnmt3b) (4.44 fold). In contrast, no significant over expression was demonstrated for transcription factor 1 (Oct3/4), activator of transcription 3 (Stat3), teratocarcinoma- derived growth factor (Tdgf1), Enk-pending (Nanog), undifferentiated embryonic cell transcription factor 1 (Utf1).

The non significant over expression of tumorigenic genes such Oct3/4, Nanog, and Utf1 in the examined hE_NSC samples might provide evidence for the non-tumorigenic nature of our examined hENSC samples and their safety for use in cell-based therapy for traumatic and neurodegenerative insults.

Recent studies have confirmed that Oct-3/4, SOX2, c-Myc, and Klf4 are essential for the process of direct reprogramming of somatic [28] and NSC to either induced pluripotent stem cells (iPS), and to induced neurons such as cholinergic and dopaminergic ones [29]. The present study demonstrated that neither Oct3/4 nor Klf4 were expressed in hENSC, SOX2, but c-Myc were significantly over expressed by 3.17, and 3.19 fold respectively. These results agree with those of Nakagawa et al. [30] who reported that mouse neural stem cells have been shown capable of reprogramming into a pluripotent state by forced expression of Oct3/4 and Klf4 only; however it is unknown whether this same strategy could apply to human NSCs, which would result in more relevant pluripotent stem cells for modeling human disease. Similar to mouse NSC, the significant over expression of SOX2, and c-Myc that the present study revealed might hint at the possibility of two factor reprogramming of human neural stem cells into pluripotency using Oct3/4 and Klf4, as has been previously demonstrated by Hester et al [31].

The expression profiles of known NSC genes were examined for all hENSC samples used in this study. The overall expression pattern of the selected genes was quite similar among the three samples tested. All hENSC samples displayed expression of the generally accepted markers of NSC Sox1, Sox2, Nestin, and Musashi1. Hundreds of genes are suggested as candidate markers for “stemness”, but their coupling to the undifferentiated stem cell state is not yet fully verified [32]. Considering the complexity of the processes involved, stemness can hardly be ensured by cooperation of just a few genes. Nevertheless, three stemness genes (namely, Oct3/4, Stat3 and Nanog) are considered “master”-genes that control the self-renewing process [33], [34].

Genome-wide gene expression profiling of an immortalized human NSC clone named HB1.F3 F3 that stably overexpressing Ngn1 identified 250 up regulated genes and 338 down regulated genes in F3-Ngn1 versus F3-WT cells [35]. Notably, the expression of LGR5, a recently identified marker for intestine and hair follicle stem cells [36], [37], [38], was greatly elevated in F3-Ngn1 cells at both mRNA and protein levels. In comparison to these findings, our DNA data had not recorded any up-regulation of Ngn1 or LGR5 in our hENSC, however, the LGR5 gene was up-regulated to 4.30 folds level in SN cells. Our data correlate with the notion that, transient overexpression of Ngn1 did not induce up regulation of LGR5 in F3-WT cells, suggesting that LGR5 is not a direct transcriptional target of Ngn1. LGR5 expression is also identified in the adult human spinal cord and brain at least at mRNA levels [39]. At present, the precise physiological function of LGR5 and downstream signaling pathways remain unknown owing to the lack of an identified natural ligand. LGR gene knockout mice showed neonatal lethality due to a breast-feeding defect caused by ankyloglossia, suggesting an involvement of LGR5 in craniofacial development [40]. A more recent study showed that LGR5 deficiency induces premature differentiation of Paneth cells in the small intestine, accompanied by overactivation of the Wnt pathway, indicating that LGR5 acts as a negative regulator of Wnt [41]. A different study revealed that LGR5 is a marker for the sublineage of intestinal stem cells that are responsive to Wnt signals derived from stem cell niche [42]. In the populations of intestinal stem cells, LGR5 labels cycling cells, while doublecortinlike kinase-1 (DCLK1) marks quiescent cells [43]. Interestingly, the expression of DCLK2, a putative paralog of DCLK1, is elevated with a 3.38-fold increase in F3-Ngn1 cells. In contrast, over expression of DCLK2 in our hENSC was not recorded.

### Correlation with other datasets

In an attempt to confirm the results obtained in the present study, we correlated the expression profiles of our studies hENSC with that of adult and fetal human neuroprogenitors available in public databases such as human hippocampal adult NPCs, human fetal NPCs from frontal cortex, multipotent adult human mesenchymal stem cells (hMSCs) as a multipotent adult stem cell control and adult (differentiated) human hippocampal tissue. Additional expression data sets in form of excel-files were obtained from the web. In accordance with the previously studied individual chips [44], [45], [46], the enrichment encountered in most of known stem cell markers that had been revealed in the present study fit well with that observed in their respective progenitor cell population providing additional validation of our protocol. Similar to our results, the human brain-derived adult neuroprogenitors cells (NPCs) showed high expression of the neural stem cell marker NES (nestin), as well as of OLIG2, MSl1, NKX2-2, and CD44. NES and MSI1 were already reported in adult NPCs by Hermann et al [47]. In addition, the expression of various SOX genes was detectable, such as SOX2 and SOX10. NTRK1 and the P75 (NGFR) genes were as well highly expressed as revealed by both the gene array as well as on RT-PCR level [47]. The fetal NPCs showed a high expression, for example, of NES, PROM1 (prominin1, CD133), Olig2, MSI1, as well as SOX genes such as SOX2, SOX3, SOX4, SOX9, and SOX11. In contrast, human mesenchymal stem cells (hMSCs) expressed various extracellular matrix proteins, such as various collagen isoform and other genes related to connective tissue and its metabolism (LOXL1, LOXL2, LUM).Fully differentiated neural tissue like the hippocampus expressed neuronal and glial genes like NTRK2, NTRK3, MBP, and GFAP [47].

Hierarchical clustering of adult NPCs and tissues containing mainly white matter, such as spinal cord and corpus callosum, is characterized by a strong expression of MHCII genes and myelin components such as peripheral myelin protein two (PMP2), myelin-associated glycoprotein (MAG), or the myelin-oligodendrocyte glycoprotein (MOG) [48]. In the present study, PMP2, PMP22 and MOG were up-regulated to 10.17, 4.43, and 9.70 fold respectively in SN neurons while they were not detected in hENSC. Interestingly, in contrast to the findings of Maisel et al [48] CD44 and GDF1/LASS1 were not expressed in our hNSCs samples. Such finding might point to unrestricted stem cell potential of our examined hENSC line in contrast to adult NPC where CD44 and GDF1/LASS1 were overexpressed [48].

Our data revealed that genes specific for differentiated neurons such as SNAP25 was markedly up-regulated to 7.49 fold in SN samples, while it was not detected in human ENSC. These findings were in harmony with that of Maisel et al [48] who demonstrated that a number of genes specific for neurons were transcribed only on reduced levels in different neuroprogenitors cells such as the 25-kDa synaptosome-associated protein (SNAP25), and neurogranin (NRGN).

In the present study, genes specifically expressed in adult NPCs such as low-affinity nerve growth factor receptor precursor (p75, NGFR), Nestin (NES), the dual specific phosphatase 10 (DUSP10), the CCmotif containing chemokine receptor seven (CCR7), the transcription factor four (TCF4), the G protein-coupled receptor 17 (GPR17), and the chondroitin sulfate proteoglycan four (CSPG4) were specifically under-expressed in our hNSC line, a findings conflicted with those of Maisel et al [48] who demonstrated a distinct over-expression of these genes.

By looking for genes that are specifically overexpressed in our hENSCs but not in SN samples, we could retrieve a large list containing genes associated with mitosis (CDC2, MCM2, PCNA, or CDKN3) or being critical for maintaining an undifferentiated state, such as SOX2, SOX3, SOX4, and SOX9. This category also includes the transcription factor eight (TCF8, also named Zfhep/deltaEF1), which was found to be expressed in early neural progenitors in the rodent brain [49]. In addition, overexpression of TCF8 was detected in vascular smooth muscle cells, human vascular endothelial cells, and in hMSCs, which points to a more general role for this gene in the development of human stem cells. In contrast to the finding Maisel et al [48] the MEIS1 gene was under-expressed in our hENSC line. Like other homeobox genes, this gene plays a crucial role in normal development. MEIS1 is especially involved in limb formation [50] but is also important for leukemic transformation [51]. A dominant-negative MEIS1 splice variant was reported to yield cell clones with impaired cell proliferation, gain of differentiated phenotype, increased contact inhibition, and cell death in human neuroblastoma lines [52]. Our MEIS1 expression pattern didn't fit that observation and suggests a non-significant role for the gene in human neural development.

We did not detect expression of more mature markers (choline acetyltransferase, tyrosine hydroxylase (TH) or those of late glial markers (glial fibrillary acidic protein, GFAP), myelin associated glycoprotein, MAG), oligodendrocyte transmembrane, OSP), suggesting that the neural cells in the sample were indeed NSC. Our hENSC samples expressed NSC genes and did not express mature neuron, oligodendrocyte, GFAP or non-neuronal lineage genes indicating absence of cell contamination.

In the present study, KEGG pathway analysis of the top 1200 unregulated genes (Log2≤5–12) of hENSC showed significant enrichment for eight molecular pathways: cell cycle, oocyte meiosis, p53 signaling pathway, progesterone-mediated oocyte maturation, viral myocarditis, ABC transporters, vibrio cholerae infection, and homologous recombination. The cell cycle pathway are dominated with genes regulating cell proliferation and mitosis such as E2F transcription factor 2, TTK protein kinase, c-abl oncogene 1, receptor tyrosine kinase, cell division cycle 2, G1 to S and G2 to M, cyclin A2, cyclin B1, cyclin B2, cyclin-dependent kinase 2, pituitary tumor-transforming 1; pituitary tumor-transforming 2, protein kinase CHK2-like; and tumor protein p53. In addition to genes involved in cell proliferation and cell division regulation, several genes related tumor transformation and tumor control were also encountered such as pituitary tumor-transforming 1 and pituitary tumor-transforming 2 and tumor protein p53. The differential expression of these genes is probably result of the embryonic nature of our examined cell lineage.

Our results could be compared to those of Yoko et al. [53] who demonstrated that genome-wide analysis of neural progenitor cells revealed 1,098 annotated mRNAs, the levels of which showed a statistically significant (P<0.05) ≥1.2-fold change in *Tis21*-GFP+S-phase NPCs as compared with *Tis21*-GFP – S-phase NPCs, with 410 mRNAs being up regulated and 688 mRNAs dow nregulated in *Tis21*-GFP+cells. Among the 1,098 genes the functions of which suggested that they may be involved in the observed cell-cycle phase alterations, notably the 3.3-fold reduction in S-phase duration (from 8.0 to 2.4 h, Table 1), in *Tis21*-GFP+compared with *Tis21*-GFP – NPCs. Indeed, alterations in mRNA levels were observed for genes involved in cell-cycle regulation and DNA replication and repair. Specifically, genes expected to be associated with a reduction in S-phase duration, such as the transcription factor E2f1 [54], [55], the phosphatase *Cdc25a* [56], *cyclin G2* [57] and *topoisomerase II alpha* [58], showed up regulated mRNA levels in *Tis21*-GFP+NPCs. Conversely, genes expected to be associated with an increase in S-phase duration, such as the repressor *E* [59], [60] and the ubiquitin ligase complex subunit Fbxw7 [61], showed downregulated mRNA levels in *Tis21*-GFP + NPCs.

Human substantia nigra was chosen because it contains a subset of dopaminergic neurons and glial cells in a mixed population and thus represents a common situation in the laboratory. Examining gene expression identified markers, such as TH, DDC, and DAT, in which the magnitude of change was sufficient to be readily assessed. We concentrated on a list of genes for dopaminergic neurons based on the published literature, using information about genes which were differentially expressed at levels that could be detected by the array. For example, LMX1B, a transcription factor required for dopaminergic neuronal development was over expressed by 2.42 fold in a human substantia nigra sample. Luo et al. [25] demonstrated that although LMX1B was not detected using a focused array in samples from human substantia nigra, this gene was detected by RT-PCR. These results indicate the reasonable sensitivity of Agilent's microarray to detect key player genes involved in the process of dopaminergic differentiation.

In many published studies, the expression of only a single (tyrosine hydroxylase, TH) or 2–3 markers for dopaminergic neurons (e.g. amino acid decarboxylase (AADC), dopamine transporter (DAT), vesicular monoamine transporter 2 (VMAT2)) have been used to indicate dopaminergic identity of neurons. In our study, several markers specific for SN samples differentiation were characterized (Tables 2, 3). Insulin-like growth factor 2 mRNA binding protein 1 was shown to be highly expressed by a 12.17 fold increase in SN samples. Other gene lists that were highly expressed in our examined samples of human SN include discs, large (Drosophila) homolog-associated protein 5 (DLGAP5), cyclin B2 (CCNB2), topoisomerase (DNA) II alpha 170 kDa (TOP2A), Holliday junction recognition protein (HJURP), sulfotransferase family, cytosolic, 6B, member 1 (SULT6B1), collagen, type II, alpha 1 (COL2A1), asp (abnormal spindle) homolog, microcephaly associated (Drosophila) (ASPM), aurora kinase B (AURKB), centromere protein A (CENPA), developmental pluripotency associated 4 (DPPA4), thymosin beta 15a; thymosin beta 15B (TMSB15A), maternal embryonic leucine zipper kinase (MELK), pentraxin-related gene, rapidly induced by IL-1 beta (PTX3), and centromere protein F, 350/400 ka (mitosin) (CENPF).

Interestingly, 18 dopamine-related genes over expressed in mesoncephalic primary culture (MesPC) of DA neurons are described as involved in cell differentiation; a subgroup of this, composed of 8 genes, is involved in neuron differentiation. Five dopamine-related MesPC genes are also associated with neurodegenerative diseases. The dopamine-related genes over-expressed in Mes. DA neurons better resampled neurophysiologic events, as they were found associated to synaptic transmission (9 genes), transmission of nerve impulse (9 genes), and behavior (7 genes) [62].

Neurogenic basic helix-loop-helix (bHLH) factors Mash1, neurogenins (Ngns) and NeuroD3 (also known as Ngn1) play important roles in Nurr1-induced mesoncephalic DA (mDA) neuronal differentiation [63]. While the role of Ngn2 in the development of mDA neurons is well known [64], [65], less information is available concerning Neurogenin [66]. In addition to inducing neurogenesis by functioning as a transcriptional activator, COUPTFII (Nr2f2) seems to be involved in tangential GABAergic interneurons migration in the developing brain, through the regulation of short- and long-range guidance cues [67]. Functions of Nr2f2 in the mDA phenotype definition are still to be described.

Our results show that the analysis of the expression arrays data using DAVID could reliably distinguish between hENSC and SN samples. It is important to emphasize that the comparison between the NSCs and the SN samples that had been conducted in the present study is not a direct precursor and differentiated cell comparison. The data presented here compares only stem cells (NSC) at a single time point in culture, undifferentiated, and a multicellular tissue from postmortem adult substantia nigra. We concentrated on the expression profiling of genes that had showed a wide range of fold increase from 2 to more than 12 folds. These include signaling molecules for Wnt-Fzd, TGF-β, Notch, fibroblast growth factor (FGF), and BMP (bone morphogenetic protein) pathways. By comparison of gene expression profiling in signaling groups between undifferentiated hENSC cells and substantia nigra samples, we have noted that some known and required pathways for induction and formation of dopaminergic neurons were activated in the SN samples. We observed enhanced expression of Nr4a2 (3.76 fold increase), En1 (6.41 fold increase), GFRA2 (5.15 fold increase) in the human substantia nigra samples that have previously been reported as signaling pathways in dopaminergic differentiation. At the same time, we could not detect any significant increase in dopaminergic genes such as Smoh, and Fzds as previously reported by Luo et al. [68]. Shh-Smoh activation and FGF8 signaling are known to be key players in midbrain pattering and genesis of dopaminergic neurons [69], [70]. Nr4a2 is a transcription factor and is expressed in both dopaminergic precursors and neurons in ventral midbrain, and deletion of Nr4a2 results in a loss of dopaminergic neurons in ventral midbrain [71], [72], [73]. Engrailed genes (En1 and En2) were shown to be involved in dopaminergic neuron survival and maintenance [74]. Activation of Wnt-Fzds pathways has multiple functions, including promoting proliferation of NSCs and dopaminergic precursors and differentiation from dopaminergic precursors to their mature neurons depending on the members of Wnts involved [75], [76], [77].

Although further dissection of signaling pathways involved in promoting dopaminergic formation is needed, our results suggest that similar pathways are activated in dopaminergic differentiation and in midbrain dopaminergic neuron formation during development such as cell adhesion molecules (CAMs), (16 genes), neuroactive ligand-receptor interaction (17 genes), tyrosine metabolism (6 genes), beta-Alanine metabolism (4 genes), tryptophan metabolism (5 genes), and calcium signaling pathway (11 genes). The cell adhesion molecules (CAMs) pathway are dominated by genes in the cluster up-regulated with dramatic fold change ranging from 5–12. The genes are involved in regulation of neuronal pre-and postsynaptic activities as well as Schwann cells and oligodendrocyte differentiation such as neural cell adhesion molecule 1, contactin 1, and contactin 2 (axonal), and claudin 11.

One focus of our study was to characterize a complex series of signaling programs potentially involved in the priming process of hNSCs. A better understanding of these signaling pathways at a cellular level would be of great assistance to the elucidation of the mechanisms underlying stem cell lineage specification and to the discovery of potential targets for stem cell therapy, gene therapy and pharmaceuticals. The signaling molecules changed during differentiation of hNSC into DA neurons were classified into 11 well-defined pathways, including G-protein signaling, Wnt, Notch, and TGF-β pathways. Notch signaling pathway has been reported widely to regulate cell fate determination, cell polarity and differentiation. Notch pathway components were regulated differently. For example, the transcription factor enhancer of split and the ligand deltex, and TACE were down regulated while CBF1 interacting corepressor was up- regulated by 2.02 fold in our hENSC samples. Notch signaling pathway has been reported widely to regulate cell fate determination, cell polarity and differentiation [55]. Notch pathway components were regulated differently. For example, the transcription factor enhancer of split and the ligand deltex, and TACE were down regulated while CBF1 interacting corepressor were up-regulated by 2.02 fold in our hENSC samples. It is believed that the Notch pathway inhibits a specific pathway of differentiation and induces differentiation along alternative pathways and that the Notch pathway is involved in asymmetric cell division and cell polarity [56]. The Wnt signaling pathway has been reported to be involved in extensive biological processes such as animal development, oncogenesis, and cell polarity establishment and so on [57]. It is widely believed that activation of the Wnt signaling pathway induces self-renewal of stem cells, and Wnt proteins act as cell growth factors [58]. Beta-catenin interacting protein one (CTNNBIP1) negatively regulates the Wnt pathway by inhibiting the interaction of beta-catenin with Tcf4 and repressing beta-catenin-Tcf4-mediated transactivation [59]. The expression level of Drctnnb1a (down-regulated by Ctnnb1) was dramatically increased by a reduction of repressing beta-catenin expression [60]. From our data, the expression of CBF1 was increased while the transcription factor enhancer of split and the ligand deltex, and TACE were down regulated in hNSC, suggesting that the Wnt signaling pathway was inhibited and that hNSCs could then gain the ability to exit the cell cycle and differentiate into neuronal cells.

In the present study, in contrast to those reported by Jun-ichi Satoh et al [36] several Wnt target genes, such as FGF [61] and Dick homolog 1 (DKK1)[43], and MMP9 [62] are coordinately down regulated, in our hENSC. FGF9 inhibits astrocyte differentiation of adult mouse NPC [63]. DKK1 is a negative regulator of Wnt signaling in HB1.F3 cells [64] MMP9 plays a central role in migration of adult NSC and NPC [65]. Moreover, Ngn1 which is also identified as a target of Wnt signaling, and it inhibits the self-renewal capacity of mouse cortical neural precursor cells [66] is not up-regulated in our hENSC. The expression of LGR5 is also controlled by the sonic hedgehog (SHH) signaling pathway [67]. SHH promotes Ngn1 expression in trigeminal neural crest cells [68]. Interestingly, both Wnt and SHH signaling pathways which are know to play a central role in NSC development and differentiation [69] were not up-regulated in our hENSC.. The down-regulation of Ngn1, and LGR5 in our examined hENSC indicate that both Wnt and SHH signaling pathways cells were not crucial for the process of proliferation, and differentiation of our examined line of hENSC. Although it remains to be investigated whether a specialized subset of LGR5 NSC exists in vivo in the adult human central nervous system (CNS).

Our data could be compared with those of Greco et al [61] who demonstrated that five functional categories were dominating among the 268 genes over-expressed in mesencephalon primary culture which are rich in DA: developmental process (95 genes), lipid metabolic process (34 genes), mitochondrion (29 genes), extracellular matrix (22 genes), and lysosome (15 genes). Additionally, neurogenesis (16 genes) and neuron differentiation (14 genes) were also significantly over-represented. Interestingly, 10 genes coding for collagens were among this group of genes with high fold change. Gene ontology (GO) classification showed significant over-representation of such families as neuron differentiation (34 genes), neurogenesis (37 genes), and neuron development (26 genes), suggesting a central role of EGR1-SP1 module during production of DA neurons.

In a recent studies, Caiazzo et al [78], and Qiang et al [79] identified a minimal set of three transcription factors—*Mash1* (also known as *Ascl1*), *Nurr1* (also known as *Nr4a2*) and *Lmx1a*—that are able to generate directly functional dopaminergic neurons from mouse and human fibroblasts without reverting to a progenitor cell stage. Our results are consistent with those of Caiazzo et al [78], and Qiang et al [79] where *Nurr1* and *Lmx1a* were up-regulated by 3.76, and 2.42 fold respectively in SN samples which is rich in DA. In contrast, *up-regulation of Mash1* (also known as *Ascl1*) was not detected.

### Conclusion

Neural stem cells (NSC) with self-renewal and multipotent properties serve as a promising cell source for transplantation to treat neurodegenerative insults such as Parkinson's disease. To efficiently induce neuronal lineage cells from NSC for dopaminergic neuron (DA) replacement therapy, we should clarify the intrinsic genetic programs involved in regulation of human NSC differentiation. We used Agilent's and Illumina's Whole Human Genome Oligonucleotide Microarray to monitor at least 41000 genes. We identified 1830 up-regulated and 1932 down-regulated mRNA in SN *vs* hE_NSC. Careful analysis of the resulting data using DAVID has permitted us to distinguish several genes and pathways between the two examined groups. We found that the set of genes expressed more highly in hENSC is enriched in molecules known or predicted to be involved in M phase of the mitotic cell cycle. On the other hand, the genes enriched at SN samples include a different set of functional categories, namely dopamine neurotransmitter release cycle, synaptic transmission, central nervous system development, structural constituents of the myelin sheath, internode region of axon, myelination, cell projection, cell soma, myelin sheath, ion transport, and the voltage-gated ion channel complex. As the SN tissue is known to be rich in DA neurons, the present results might provide insights to identify the key regulators and biomarkers that may allow targeted selection of a limited number of genes or transcription factors to be used for direct reprogramming of NSC into DA cells with an ultimate goal of obtaining different types of allogenic neurons including personalized DA neurons.

## Supporting Information

Table S1 Top 10 probe-sets showing largest fold-difference in expression (hENSCs) and SN.
(DOC)

Table S2 Genomic markers related to dopaminergic system.
(DOC)

## References

[1] Emsley JG, Mitchell BD, Kempermann G, Macklis JD (2005). Adult neurogenesis and repair of the adult CNS with neural progenitors, precursors, and stem cells.. Prog Neurobiol.

[2] Lichtenwalner RJ, Parent JM (2006). Adult neurogenesis and the ischemic forebrain.. J Cereb Blood Flow Metab.

[3] Thomson JA, Itskovitz-Eldor J, Shapiro SS (1998). Embryonic stem cell lines derived from human blastocysts.. Science.

[4] Mitalipova M, Calhoun J, Shin S (2003). Human embryonic stem cell lines derived from discarded embryos.. STEM CELLS.

[5] Wagner J, Akerud P, Castro DS, Holm PC, Canals JM (1999). Induction of a midbrain dopaminergic phenotype in Nurr1- overexpressing neural stem cells by type 1 astrocytes.. Nat Biotechnol.

[6] Wang X, Lu Y, Zhang H, Wang K, He Q (2004). Distinct efficacy of pre-differentiated versus intact fetal mesencephalon-derived human neural progenitor cells in alleviating rat model of Parkinson's disease.. Int J Dev Neurosci.

[7] Liste I, García-García E, Martínez-Serrano A (2004). The generation of dopaminergic neurons by human neural stem cells is enhanced by Bcl-XL, both in vitro and in vivo.. J Neurosci.

[8] Carvey PM, Ling ZD, Sortwell LE, Pitzer MR, McGuire SO (2001). A clone line of mesencephalic progenitor cells converted to dopamine neurons by hematopoietic cytokines: a source of cells for transplantation in Parkinson's disease.. Exp Neural.

[9] Lee SH, Lumelsky N, Studer L (2000). Efficient generation of midbrain and hindbrain neurons from mouse embryonic stem cells.. Nat Biotechnol.

[10] Chung S, Hedlund E, Hwang M (2005). The homeodomain transcription factor Pitx3 facilitates differentiation of mouse embryonic stem cells into AHD2-expressing dopaminergic neurons.. Mol Cell Neurosci.

[11] Zeng X, Cai J, Chen J (2004). Dopaminergic differentiation of human embryonic stem cells.. STEM CELLS.

[12] Park CH, Minn YK, Lee JY (2005). In vitro and in vivo analyses of human embryonic stem cell-derived dopamine neurons.. J Neurochem.

[13] Takahashi K, Tanabe K, Ohnuki M (2007). Induction of pluripotent stem cells from adult human fibroblasts by defined factors.. Cell.

[14] Reynolds BA, Weiss S (1996). Clonal and population analyses demonstrate that an EGF-responsive mammalian embryonic CNS precursor is a stem cell.. Dev Biol.

[15] Ashburner M, Ball CA, Blake JA, Botstein D, Butler H (2000). Gene ontology: Tool for the unification of biology. The gene ontology consortium.. Nat Genet.

[16] Yang YH, Dudoit S, Luu P (2001). Normalization for cDNA microarray data.. http://www.stat.berkeley.edu/users/terry/zarray/Html/normspie.html.

[17] Huang DW, Sherman BT, Lempicki RA (2009). Systematic and integrative analysis of large gene lists using DAVID Bioinformatics Resources.. Nature Protoc.

[18] Dennis G, Sherman BT, Hosack DA, Yang J, Gao W (2003). DAVID: Database for Annotation, Visualization, and Integrated Discovery.. Genome Biol.

[19] Su AI, Cooke MP, Ching KA (2002). Large-scale analysis of the human and mouse transcriptomes.. Proc Natl Acad Sci U S A.

[20] Ge X, Yamamoto S, Tsutsumi S (2005). Interpreting expression profiles of cancers by genome-wide survey of breadth of expression in normal tissues.. Genomics.

[21] Irizarry RA, Hobbs B, Collin F (2003). Exploration, normalization, and summaries of high density oligonucleotide array probe level data.. Biostatistics.

[22] Taupin P, Ray J, Fischer WH, Suhr ST, Hakansson K (2000). FGF-2-responsive neural stem cell proliferation requires CCg, a novel autocrine/paracrine cofactor.. Neuron.

[23] Schein JC, Hunter DD, Roffler-Tarlov S (1998). Girk2 expression in the ventral midbrain, cerebellum, and olfactory bulb and its relationship to the murine mutation weaver.. Dev Biol.

[24] Yamada T, McGeer PL, Baimbridge KG (1990). Relative sparing in Parkinson's disease of substantia nigra dopamine neurons containing calbindin-D28K.. Brain Res.

[25] Gibb WR (1992). Melanin, tyrosine hydroxylase, calbindin and substance P in the human midbrain and substantia nigra in relation to nigrostriatal projections and differential neuronal susceptibility in Parkinson's disease.. Brain Res.

[26] German DC, Manaye K, Smith WK (1989). Midbrain dopaminergic cell loss in Parkinson's disease: computer visualization.. Ann Neurol.

[27] Bhattacharya B, Miura T, Brandenberger R, Mejido J, Luo Y (2004). Gene expression in human embryonic stem cell lines: unique molecular signature.. Blood.

[28] Takahashi K, Yamanaka S (2006). Induction of pluripotent stem cells from mouse embryonic and adult fibroblast cultures by defined factors.. Cell.

[29] Swistowski A, Peng J, Liu Q, Mali P, Rao MS (2010). Efficient generation of functional dopaminergic neurons from human induced pluripotent stem cells under defined conditions.. Stem Cells.

[30] Nakagawa M, Koyanagi M, Tanabe K, Takahashi K, Ichisaka T (2008). Generation of induced pluripotent stem cells without Myc from mouse and human fibroblasts.. Nat Biotechnol.

[31] Hester ME, Song S, Miranda CJ, Eagle A, Schwartz PH (2009). Two Factor Reprogramming of Human Neural Stem Cells into Pluripotency.. PLoS ONE.

[32] Cai J, Weiss ML, Rao MS (2004). In search of “stemness”.. Exp Hematol.

[33] Chambers I, Colby D, Robertson M, Nichols J, Lee S (2003). Functional expression cloning of Nanog, a pluripotency sustaining factor in embryonic stem cells.. Cell.

[34] Mitsui K, Tokuzawa Y, Itoh H, Segawa K, Murakami M (2003). The homeoprotein Nanog is required for maintenance of pluripotency in mouse epiblast and ES cells.. Cell.

[35] Satoh J, Obayashi S, Tabunoki H, Wakana T, Kim SU (2010). Stable Expression of Neurogenin 1 Induces LGR5, a Novel Stem Cell Marker, in an Immortalized Human Neural Stem Cell Line HB1.F3.. Cell Mol Neurobiol.

[36] Barker N, van Es JH, Kuipers J, Kujala P, van den Born M (2007). Identification of stem cells in small intestine and colon by marker gene Lgr5.. Nature.

[37] Jaks V, Barker N, Kasper M, van Es JH, Snippert HJ (2008). Lgr5 marks cycling, yet long-lived, hair follicle stem cells.. Nat Genet.

[38] Sato H, Ishida S, Toda K, Matsuda R, Hayashi Y (2005). New approaches to mechanism analysis for drug discovery using DNA microarray data combined with KeyMolnet.. Curr Drug Discov Technol.

[39] Hsu SY, Liang SG, Hsueh AJ (1998). Characterization of two LGR genes homologous to gonadotropin and thyrotropin receptors with extracellular leucine-rich repeats and a G protein-coupled, seven-transmembrane region.. Mol Endocrinol.

[40] Morita H, Mazerbourg S, Bouley DM, Luo CW, Kawamura K (2004). Neonatal lethality of LGR5 null mice is associated with ankyloglossia and gastrointestinal distension.. Mol Cell Biol.

[41] Garcia MI, Ghiani M, Lefort A, Libert F, Strollo S (2009). LGR5 deficiency deregulates Wnt signaling and leads to precocious Paneth cell differentiation in the fetal intestine.. Dev Biol.

[42] May R, Sureban SM, Hoang N, Riehl TE, Lightfoot SA (2009). DCAMKL-1 and LGR5 mark quiescent and cycling intestinal stem cells respectively.. Stem Cells.

[43] Niida A, Hiroko T, Kasai M, Furukawa Y, Nakamura Y (2004). DKK1, a negative regulator of Wnt signaling, is a target of the b-catenin/TCF pathway.. Oncogene.

[44] Su AI, Cooke MP, Ching KA (2002). Large-scale analysis of the human and mouse transcriptomes.. Proc Natl Acad Sci U S A.

[45] Ge X, Yamamoto S, Tsutsumi S (2005). Interpreting expression profiles of cancers by genome-wide survey of breadth of expression in normal tissues.. Genomics.

[46] Irizarry RA, Hobbs B, Collin F (2003). Exploration, normalization, and summaries of high density oligonucleotide array probe level data.. Biostatistics.

[47] Hermann A, Maisel M, Liebau S (2006). Mesodermal cell types induce neurogenesis from adult human hippocampal progenitor cells.. J Neurochem.

[48] Maisel M, Herr A, Milosevic J, Hermann A, Habisch H (2007). Transcription Profiling of Adult and Fetal Human Neuroprogenitors Identifies Divergent Paths to Maintain the Neuroprogenitor Cell State.. STEM CELLS.

[49] Yen G, Croci A, Dowling A (2001). Developmental and functional evidence of a role for Zfhep in neural cell development.. Brain Res Mol Brain Res.

[50] Mercader N, Leonardo E, Azpiazu N (1999). Conserved regulation of proximodistal limb axis development by Meis1/Hth.. Nature.

[51] Thorsteinsdottir U, Kroon E, Jerome L (2001). Defining roles for HOX and MEIS1 genes in induction of acute myeloid leukemia.. Mol Cell Biol.

[52] Geerts D, Schilderink N, Jorritsma G (2003). The role of the MEIS homeobox genes in neuroblastoma.. Cancer Lett.

[53] Yoko A, Pulvers JN, Haffner C, Schilling B, Nüsslein I (2011). Neural stem and progenitor cells shorten S-phase on commitment to neuron production.. nature Communications.

[54] Mitsui K, Tokuzawa Y, Itoh H, Segawa K, Murakami M (2003). The homeoprotein Nanog is required for maintenance of pluripotency in mouse epiblast and ES cells.. Cell.

[55] Ohishi K, Varnum-Finney B, Bernstein ID (2002). The notch pathway: modulation of cell fate decisions in hematopoiesis.. Int J Hematol.

[56] Ohishi K, Katayama N, Shiku H, Varnum-Finney B, Bernstein ID (2003). Notch signalling in hematopoiesis.. Semin Cell Dev Biol.

[57] van de Wetering M, de Lau W, Clevers H (2002). WNT signaling and lymphocyte development.. Cell.

[58] Willert K, Brown JD, Danenberg E, Duncan AW, Weissman IL (2003). Wnt proteins are lipid-modified and can act as stem cell growth factors.. Nature.

[59] Tago K, Nakamura T, Nishita M, Hyodo J, Nagai S (2000). Inhibition of Wnt signaling by ICAT, a novel beta-catenin-interacting protein.. Genes Dev.

[60] Kawasoe T, Furukawa Y, Daigo Y, Nishiwaki T, Ishiguro H (2000). Isolation and characterization of a novel human gene, DRCTNNB1A, the expression of which is down-regulated by betacatenin.. Cancer Res.

[61] Hendrix ND, Wu R, Kuick R, Schwartz DR, Fearon ER (2006). Fibroblast growth factor 9 has oncogenic activity and is a downstream target of Wnt signaling in ovarian endometrioid adenocarcinomas.. Cancer Res.

[62] Wu B, Crampton SP, Hughes CC (2007). Wnt signaling induces matrix metalloproteinase expression and regulates T cell transmigration.. Immunity.

[63] Lum M, Turbic A, Mitrovic B, Turnley AM (2009). Fibroblast growth factor-9 inhibits astrocyte differentiation of adult mouse neural progenitor cells.. J Neurosci Res.

[64] Ahn SM, Byun K, Kim D, Lee K, Yoo JS (2008). Olig2-induced neural stem cell differentiation involves downregulation of Wnt signaling and induction of Dickkopf-1 expression.. PLoS One.

[65] Barkho BZ, Munoz AE, Li X, Li L, Cunningham LA (2008). Endogenous matrix metalloproteinase (MMP)-3 and MMP-9 promote the differentiation and migration of adult neural progenitor cells in response to chemokines.. Stem Cells.

[66] Hirabayashi Y, Itoh Y, Tabata H, Nakajima K, Akiyama T (2004). The Wnt/b-catenin pathway directs neuronal differentiation of cortical neural precursor cells.. Development.

[67] Tanese K, Fukuma M, Yamada T, Mori T, Yoshikawa T (2008). G-protein-coupled receptor GPR49 is up-regulated in basal cell carcinoma and promotes cell proliferation and tumor formation.. Am J Pathol.

[68] Ota M, Ito K (2003). Induction of neurogenin-1 expression by sonic hedgehog: its role in development of trigeminal sensory neurons.. Dev Dyn.

[69] Prakash N, Wurst W (2007). A Wnt signal regulates stem cell fate and differentiation in vivo.. Neurodegener Dis.

[70] Hynes M, Rosenthal A (1999). Specification of dopaminergic and serotonergic neurons in the vertebrate CNS.. Curr Opin Neurobio.

[71] Zetterstrom RH, Solomin L, Jansson L (1977). Dopamine neuron agenesis in Nurr1-deficient mice.. Science.

[72] Saucedo-Cardenas O, Quintana-Hau JD, Le WD (1998). Nurr1 is essential for the induction of the dopaminergic phenotype and the survival of ventral mesencephalic late dopaminergic precursor neurons.. Proc Natl Acad Sci U S A.

[73] Castillo SO, Baffi JS, Palkovits M (1998). Dopamine biosynthesis is selectively abolished in substantia nigra/ventral tegmental area but not in hypothalamic neurons in mice with targeted disruption of the Nurr1 gene.. Mol Cell Neurosci.

[74] Simon HH, Saueressig H, Wurst W (2001). Fate of midbrain dopaminergic neurons controlled by the engrailed genes.. J Neurosci.

[75] Thomas KR, Capecchi MR (1990). Targeted disruption of the murine int-1 proto-oncogene resulting in severe abnormalities in midbrain and cerebellar development.. Nature.

[76] Panhuysen M, Vogt Weisenhorn DM, Blanquet V (2004). Effects of Wnt1 signaling on proliferation in the developing mid-/hindbrain region.. Mol Cell Neurosci.

[77] Castelo-Branco G, Wagner J, Rodriguez FJ (2003). Differential regulation of midbrain dopaminergic neuron development by Wnt-1, Wnt-3a, and Wnt-5a.. Proc Natl Acad Sci U S A.

[78] Massimiliano C, Maria T, Elena D, Dejan L, Stefano T (2011). Direct generation of functional dopaminergic neurons from mouse and human fibroblasts.. Nature.

[79] Qiang L, Fujita R, Yamashita T, Angulo S, Rhinn H (2011). Directed Conversion of Alzheimer's Disease Patient Skin Fibroblasts into Functional Neurons.. Cell.

