# Supplementary material for: Gene Expression Profiling of Embryonic Human Neural Stem Cells and Dopaminergic Neurons from Adult Human Substantia Nigra
Source: PLoS One. 2011 Dec 7;6(12):e28420. doi: 10.1371/journal.pone.0028420 (PMC3233561; doi:10.1371/journal.pone.0028420)
Supplement: Table S1 — Top 10 probe-sets showing largest fold-difference in expression (hENSCs) and SN. (DOC) [file pone.0028420.s001.doc]

| Supplementary Table 1. Top 10 probe-sets showing largest fold-difference in expression (hENSCs) and DA. | | | | |
| --- | --- | --- | --- | --- |
| **Top 10 probe-sets showing largest fold-difference in expression (hENSCs)** | | | | |
| **Probe ID** | **Gene Symbol** | **Fold Change** | **Description** | **Functional Classification GO group and** |
| A_23_P83498 | IGF2BP1 | -12.17 | insulin-like growth factor 2 mRNA binding protein 1 | GO:0000166: nucleotide binding, GO:0003723: RNA binding, GO:0005515: protein binding, GO:0005634: nucleus, GO:0005737: cytoplasm, GO:0017148: negative regulation of translation, GO:0042035: regulation of cytokine biosynthetic process, GO:0045182: translation regulator activity, GO:0048027: mRNA 5'-UTR binding |
| A_23_P95930 | HMGA2 | -12.17 | high mobility group AT-hook 2 | GO:0000228: nuclear chromosome, GO:0000785: chromatin, GO:0003677: DNA binding, GO:0003680: AT DNA binding, GO:0005515: protein binding, GO:0005634: nucleus, GO:0006325: establishment or maintenance of chromatin architecture, GO:0006350: transcription, GO:0006355: regulation of transcription, DNA-dependent, GO:0007049: cell cycle, GO:0007067: mitosis, GO:0007275: multicellular organismal development, GO:0040008: regulation of growth, GO:0051301: cell division |
| A_23_P88331 | DLGAP5 | -11.55 | discs, large (Drosophila) homolog-associated protein 5 | GO:0000087: M phase of mitotic cell cycle, GO:0004721: phosphoprotein phosphatase activity, GO:0005515: protein binding, GO:0005634: nucleus, GO:0005737: cytoplasm, GO:0007049: cell cycle, GO:0007079: mitotic chromosome movement towards spindle pole, GO:0007267: cell-cell signaling, GO:0008283: cell proliferation, GO:0031616: spindle pole centrosome, GO:0045842: positive regulation of mitotic metaphase/anaphase transition |
| A_32_P62997 | PBK | -10.97 | PDZ binding kinase | GO:0000166: nucleotide binding, GO:0004674: protein serine/threonine kinase activity, GO:0005515: protein binding, GO:0005524: ATP binding, GO:0005575: cellular_component, GO:0006468: protein amino acid phosphorylation, GO:0007067: mitosis, GO:0016740: transferase activity |
| A_23_P118834 | TOP2A | -10.66 | topoisomerase (DNA) II alpha 170kDa | GO:0000166: nucleotide binding, GO:0003682: chromatin binding, GO:0003918: DNA topoisomerase (ATP-hydrolyzing) activity, GO:0005080: protein kinase C binding, GO:0005524: ATP binding, GO:0005634: nucleus, GO:0005654: nucleoplasm, GO:0005694: chromosome, GO:0005730: nucleolus, GO:0005737: cytoplasm, GO:0005814: centriole, GO:0006260: DNA replication, GO:0006265: DNA topological change, GO:0006266: DNA ligation, GO:0006281: DNA repair, GO:0006974: response to DNA damage stimulus, GO:0007059: chromosome segregation, GO:0008022: protein C-terminus binding, GO:0008094: DNA-dependent ATPase activity, GO:0008144: drug binding, GO:0009330: DNA topoisomerase complex (ATP-hydrolyzing), GO:0019035: viral integration complex, GO:0030263: apoptotic chromosome condensation, GO:0042803: protein homodimerization activity, GO:0042826: histone deacetylase binding, GO:0043065: positive regulation of apoptosis, GO:0043130: ubiquitin binding, GO:0045870: positive regulation of retroviral genome replication, GO:0046982: protein heterodimerization activity, GO:0048015: phosphoinositide-mediated signaling |
| A_23_P65757 | CCNB2 | -10.58 | cyclin B2 | GO:0001701: in utero embryonic development, GO:0005515: protein binding, GO:0005624: membrane fraction, GO:0005634: nucleus, GO:0005829: cytosol, GO:0007049: cell cycle, GO:0007067: mitosis, GO:0015630: microtubule cytoskeleton, GO:0040007: growth, GO:0043029: T cell homeostasis, GO:0048538: thymus development, GO:0051301: cell division |
| A_24_P297539 | UBE2C | -10.54 | ubiquitin-conjugating enzyme E2C | GO:0004842: ubiquitin-protein ligase activity, GO:0005524: ATP binding, GO:0005654: nucleoplasm, GO:0005829: cytosol, GO:0006511: ubiquitin-dependent protein catabolic process, GO:0007049: cell cycle, GO:0007051: spindle organization, GO:0007067: mitosis, GO:0008054: cyclin catabolic process, GO:0016567: protein ubiquitination, GO:0016874: ligase activity, GO:0031145: anaphase-promoting complex-dependent proteasomal ubiquitin-dependent protein catabolic process, GO:0031536: positive regulation of exit from mitosis, GO:0048015: phosphoinositide-mediated signaling, GO:0051246: regulation of protein metabolic process, GO:0051301: cell division, GO:0051436: negative regulation of ubiquitin-protein ligase activity during mitotic cell cycle, GO:0051437: positive regulation of ubiquitin-protein ligase activity during mitotic cell cycle |
| A_33_P3807062 | HJURP | -10.50 | Holliday junction recognition protein | GO:0003677: DNA binding, GO:0005634: nucleus |
| A_23_P500464 | COL2A1 | -10.47 | collagen, type II, alpha 1 | GO:0005576: extracellular region, GO:0005581: collagen, GO:0005585: collagen type II, GO:0007601: visual perception, GO:0007605: sensory perception of sound, GO:0030020: extracellular matrix structural constituent conferring tensile strength, GO:0030199: collagen fibril organization, GO:0042802: identical protein binding, GO:0051216: cartilage development, GO:0060272: embryonic skeletal joint morphogenesis |
| A_23_P52017 | ASPM | -10.46 | asp (abnormal spindle) homolog, microcephaly associated | GO:0005516: calmodulin binding, GO:0005634: nucleus, GO:0005737: cytoplasm, GO:0007049: cell cycle, GO:0007067: mitosis, GO:0051301: cell division |
| **Top 10 probe-sets showing largest fold-difference in expression (DA cells).** | | | | |
| **Probe ID** | **Gene Symbol** | **Fold Change** | **Description** | **Functional Classification GO group and** |
| A_33_P3363804 | NCAM1 | 14.16 | neural cell adhesion molecule 1 | GO:0005515: protein binding, GO:0005576: extracellular region, GO:0005886: plasma membrane, GO:0007155: cell adhesion, GO:0016021: integral to membrane, GO:0031225: anchored to membrane |
| A_33_P3227990 | MBP | 14.06 | myelin basic protein | GO:0005886: plasma membrane, GO:0006955: immune response, GO:0007268: synaptic transmission, GO:0007417: central nervous system development, GO:0019911: structural constituent of myelin sheath, GO:0033269: internode region of axon, GO:0042552: myelination, GO:0042995: cell projection, GO:0043025: cell soma, GO:0043209: myelin sheath |
| A_23_P49448 | FA2H | 13.88 | fatty acid 2-hydroxylase | GO:0005783: endoplasmic reticulum, GO:0005789: endoplasmic reticulum membrane, GO:0005792: microsome, GO:0006633: fatty acid biosynthetic process, GO:0006665: sphingolipid metabolic process, GO:0006810: transport, GO:0016020: membrane, GO:0016021: integral to membrane, GO:0016491: oxidoreductase activity, GO:0020037: heme binding, GO:0022900: electron transport chain, GO:0046872: metal ion binding |
| A_23_P212508 | TF | 13.28 | transferrin | GO:0001666: response to hypoxia, GO:0005576: extracellular region, GO:0005604: basement membrane, GO:0005615: extracellular space, GO:0005739: mitochondrion, GO:0005768: endosome, GO:0006811: ion transport, GO:0006879: cellular iron ion homeostasis, GO:0006953: acute-phase response, GO:0008199: ferric iron binding, GO:0014070: response to organic cyclic substance, GO:0015091: ferric iron transmembrane transporter activity, GO:0015682: ferric iron transport, GO:0030139: endocytic vesicle, GO:0031643: positive regulation of myelination, GO:0046872: metal ion binding |
| A_23_P26457 |  |  |  |  |
| A_23_P353035 | IGFBP7 | 12.56 | insulin-like growth factor binding protein 7 | GO:0001558: regulation of cell growth, GO:0005520: insulin-like growth factor binding, GO:0005576: extracellular region, GO:0007155: cell adhesion, GO:0008285: negative regulation of cell proliferation |
| A_23_P171074 | ITM2A | 12.21 | integral membrane protein 2A | GO:0005737: cytoplasm, GO:0005886: plasma membrane, GO:0016021: integral to membrane |
| A_32_P187571 | SCN2B | 12.05 | sodium channel, voltage-gated, type II, beta | GO:0001518: voltage-gated sodium channel complex, GO:0005244: voltage-gated ion channel activity, GO:0005248: voltage-gated sodium channel activity, GO:0006811: ion transport, GO:0006814: sodium ion transport, GO:0007268: synaptic transmission, GO:0016020: membrane, GO:0016021: integral to membrane, GO:0031402: sodium ion binding |
| A_23_P66241 | MT1M | 11.97 | metallothionein 1M | GO:0005507: copper ion binding, GO:0008270: zinc ion binding, GO:0046870: cadmium ion binding, GO:0046872: metal ion binding |
| A_23_P9869 | CNDP1 | 11.92 | carnosine dipeptidase 1 (metallopeptidase M20 family) | GO:0004180: carboxypeptidase activity, GO:0005576: extracellular region, GO:0006508: proteolysis, GO:0008233: peptidase activity, GO:0008237: metallopeptidase activity, GO:0008270: zinc ion binding, GO:0046872: metal ion binding, GO:0046983: protein dimerization activity |
