# Supplementary material for: Gene Expression Profiling of Embryonic Human Neural Stem Cells and Dopaminergic Neurons from Adult Human Substantia Nigra
Source: PLoS One. 2011 Dec 7;6(12):e28420. doi: 10.1371/journal.pone.0028420 (PMC3233561; doi:10.1371/journal.pone.0028420)
Supplement: Table S2 — Genomic markers related to dopaminergic system. (DOC) [file pone.0028420.s002.doc]

| Supplementary Table 2: Genomic markers related to dopaminergic system. | |
| --- | --- |
| Genes and folds increase in DA cells | Function |
| Igf1 (4.73 folds), Th (5.91 folds) | Markers of dopaminergic neurons |
| En1 (6.41 folds), WNT10B (2.5 folds), WNT11 (4.35) | Markers of dopaminergic progenitors |
| Lhx1 (Lim1) (4.52 folds) | Markers of dopaminergic progenitors maturation |
| Gfra1 (1.56 folds), Gfra2 (5.15 folds), Otx2 (3.53 folds) | Markers of early dopaminergic neurons |
| Adcy7 (2.08 folds), Alcam (3.72 folds), Bcl11a (3.33 folds), Cart (6.36 folds), Cbln1 (6.31 folds), Drd5 (1.38 folds), Egr1 (1.24 folds), Fos (1.31 folds), Ghr (3.54 folds), Grin2c (5.76 folds), Grp (5.06 folds), Kcna5 (6.74 folds), Kcnab1 (3.54 folds), Mlp (Marcksl1) (2.57 folds), Moxd1 (2.01 folds), Mpp3 (1.25 folds), Nrip3 (4.89 folds), Nrp2 (2.02 folds). | Related to dopaminergic system |
